# Mystique, a broad host range *Acinetobacter* phage, reveals the impact of culturing conditions on phage isolation and infectivity

**DOI:** 10.1371/journal.ppat.1012986

**Published:** 2025-04-10

**Authors:** Ellinor O Alseth, Carli Roush, Iris Irby, Mykhailo Kopylov, Daija Bobe, Monneh W Diggs, Kristy Nguyen, Huaijin Xu, Ingeborg Schmidt-Krey, Anton V Bryksin, Philip N Rather

**Affiliations:** 1 Center for Microbial Dynamics and Infection, Georgia Institute of Technology, Atlanta, Georgia, United States of America; 2 School of Biological Sciences, Georgia Institute of Technology, Atlanta, Georgia, United States of America; 3 New York Structural Biology Center, New York, New York, United States of America; 4 School of Chemistry & Biochemistry, Georgia Institute of Technology, Atlanta, Georgia, United States of America; 5 Molecular Evolution Core Facility, Georgia Institute of Technology, Atlanta, Georgia, United States of America; 6 Department of Microbiology and Immunology, Emory University, Atlanta, Georgia, United States of America; 7 Atlanta VA Healthcare System, Decatur, Georgia, United States of America; Monash University, AUSTRALIA

## Abstract

With the global rise of antimicrobial resistance, phage therapy is increasingly re-gaining traction as a strategy to treat bacterial infections. For phage therapy to be successful however, we first need to isolate appropriate candidate phages for both clinical and experimental research*. Acinetobacter baumannii* is an opportunistic pathogen known for its ability to rapidly evolve resistance to antibiotics, making it a prime target for phage therapy. Yet phage isolation may be hampered by *A. baumannii*’s ability to rapidly switch between capsular states. Here, we report the discovery and structural characterisation of a novel lytic phage, Mystique. This phage was initially isolated against the wild-type AB5075: a commonly used clinical model strain. When screening Mystique on 103 highly diverse isolates of *A. baumannii*, we found that it has a broad host range, being able to infect 85.4% of all tested strains when tested on bacterial lawns – a host range that expanded to 91.3% when tested in liquid culture. This variation between solid and liquid culturing conditions on phage infectivity was also observed for several other phages in our collection that were assumed unable to infect AB5075, and some capsule negative mutants that seemed resistant to Mystique proved susceptible when assayed in liquid. This highlights how differences in culturing conditions can drastically impact phage infectivity, with important consequences for phage isolation and characterisation efforts. Finally, Mystique was found to be able to infect other species of *Acinetobacter*, making it a multi-species phage with broad applicability for further research.

## Author summary

Bacterial infections caused by *Acinetobacter baumannii* are a major global health concern due to high antibiotic resistance, earning it a critical priority pathogen ranking by the WHO. Phage therapy is resurging as a treatment option, with some success against *A. baumannii*. However, the important clinical model strain AB5075 lacks a readily available and characterised phage, and isolating phages for *A. baumannii* is challenging due to its diverse capsule. Here, we report the discovery of a novel lytic phage, Mystique, which exhibits a broad host range, infecting 94 out of 103 tested *A. baumannii* strains, and three other *Acinetobacter* species. We conducted genomic sequencing and structural analysis to fully characterise Mystique, as well as finding that disrupting the host’s capsule synthesis process limits phage infectivity. Additionally, we found that culturing conditions significantly impact results; some phages that do not form plaques on bacterial lawns can still infect and amplify in liquid cultures of the same strain. Moreover, mutants resistant to Mystique based on plaque assays were susceptible in liquid culture assays. This work underscores the necessity of a multifaceted approach for phage research, as traditional phage assays may not be sufficient for studying bacteria-phage dynamics in certain bacteria such as *Acinetobacter*.

## Introduction

*Acinetobacter baumannii* is an increasingly antibiotic resistant and virulent bacterium, known to cause severe nosocomial infections [1–4]. With an estimated 63% of isolates in the United States being considered multidrug resistant [5], infections due to *A. baumannii* are difficult to overcome and often fatal [6]. An additional challenge is *A. baumannii’*s ability to contaminate and persist in healthcare facilities, such as in laminar flow systems [7], on care and medical equipment [8–10], and on other surfaces like curtains, door handles, and keyboards [10,11]. It is largely due to these challenges of preventing and treating *A. baumannii* infections that the therapeutic application of bacteriophages (phages, *i*.*e*. viruses that infect bacteria) is increasingly being considered and – usually as a last resort – applied [12,13].

To improve the efficacy of phage therapy, we first need to improve our understanding of bacteria-phage dynamics using microbial model systems (which in general are crucial in advancing science across various biological disciplines [14,15]). Bacteria-phage model systems provide researchers with a highly controlled environment for testing experimental predictions that may elucidate fundamental principles underlying bacteria-phage interactions, such as mechanisms of phage infection [16], phage resistance [17], and coevolutionary dynamics [18]. Additionally, bacteria-phage model systems serve as helpful tools when exploring the application of phages as therapeutics and their potential consequences [19]. After all, the arms race between phage (‘predator’) and bacteria (‘prey’) is fast-paced, with a strong selection pressure for the bacteria being targeted to evolve phage resistance both *in vitro* and *in vivo* [20]. Microbial model systems for studying bacteria-phage interactions have been developed for other opportunistic pathogens such as *Escherichia coli* [21,22] and *Pseudomonas aeruginosa* [23,24], yet no well-characterised phage is readily available for some clinically relevant strains of *A. baumannii*. This is the case for the wild-type clinical isolate and model strain AB5075 in particular. AB5075 is a highly virulent strain of *A. baumannii* that is commonly used in various animal models to study pathogenesis, host-pathogen interactions, and to assess potential new treatments [25]. Two phages were previously reported to have been isolated against capsulated AB5075 (capsule locus KL25 [26]), but have not been made available to researchers, have not been characterised beyond initial isolation, or both [27,28].

In an otherwise genetically homogenous population, AB5075 and other *A. baumannii* strains exhibit phenotypic heterogeneity by rapidly switching between virulent opaque (VIR-O) and avirulent translucent (AV-T) colonies [29–31]. This phenotypic switch is associated with changes in capsule thickness, with AV-T cells exhibiting a twofold decrease in capsule thickness compared to VIR-O cells [30]. While the switching frequency is at ~4-13% over 24 hours for a single propagated colony of AB5075 [30], this rate is potentially affected by the selection pressure imposed by the presence of phages targeting one state but not the other. In other words, the process of isolating novel phages can incur an increased selection pressure for rapid capsule modulation at a rate incompatible with commonly applied methods of phage isolation. For *A. baumannii*, many phages have been found to select for reduced capsule production [32,33], but most phage isolation attempts for AB5075 specifically have resulted in phages targeting capsule negative mutants [27,34], but rarely the wild-type. It is therefore possible that a thickening of the capsule might directly block or otherwise hinder phage absorption in some strains of *A. baumannii*; a characteristic found in other bacteria with similar capsule properties [35].

Here, we report our successful phage isolation for AB5075, resulting in the isolation of Mystique: a novel lytic *Acinetobacter* phage. In addition to AB5075, Mystique can infect 91.3% of a diverse set (*n* = 103, including AB5075) of clinical *A. baumannii* isolates. This makes for a remarkably broad host range, especially as the strains Mystique was tested on are specifically meant to represent the genetic diversity of *A. baumannii* as a species [36]. Further, we found that Mystique can infect other species of *Acinetobacter*, namely *Acinetobacter nosocomialis*, *Acinetobacter calcoaceticus*, and *Acinetobacter baylyi*, making it a multi-species *Acinetobacter* phage. To isolate phage Mystique, we combined raw sewage with a cocktail of known phages that can infect but not plaque on AB5075, to potentially limit resistance evolution occurring during the isolation process. The isolation and characterisation process for this phage also revealed how assays done on agar plates can lead to false negative results when testing phage infectivity, as we discovered multiple phages in our collection were able to infect AB5075 in liquid culture but not on a bacterial lawn. Additionally, some capsule mutants of AB5075 that would traditionally be classified as resistant to Mystique based on plaque assays proved susceptible to the phage in liquid cultures. This likely has important implications for any phage isolation attempt for *A. baumannii* and other bacteria with similar capsule properties, seeing as most phage assays are performed on bacterial lawns: a method that has not seen much change since the discovery of phages in the early 1900s [37,38].

## Results

### Isolation of novel *Acinetobacter* phage Mystique using a phage-cocktail approach

There is currently no well-characterised lytic phage readily available against the wild-type clinical model strain of *A. baumannii* AB5075, and yet a phage is essential to study clinically relevant bacteria-phage dynamics. To solve this issue, we isolated a novel lytic phage, named Mystique, from local sewage water in Atlanta, Georgia, USA, using AB5075 as the bacterial target for phage infection. All the experiments described below were done with the VIR-O form of AB5075 unless stated otherwise.

Initially, however, the isolation of phage Mystique seemed to have been in vain, as no phage plaques were observed on a lawn of AB5075 after our first isolation attempt. Capsular phase variation has previously been shown to be a driver for phage persistence in other bacteria, by allowing for dynamic sub-populations of sensitive and resistant host cells [39]. Based on this and *A. baumannii* phages often being highly specific in regards to the capsular polysaccharides they target [32], we hypothesised that the rapid phenotypic switching between VIR-O and AV-T states as a potential strategy to become phage resistant could be a hindrance in the initial phage amplification step. In an attempt to limit the evolution of phage resistance, we then speculated that the addition of other phages could constrain the evolution of phage resistance.

In our phage biobank we have three phages (Maestro [20], FG03, and CO01 [33]) that plaque on *A. baumannii* MRSN 423159 [36], a strain which in general seems to be broadly susceptible to multiple phages. MRSN 423159 has capsule locus type KL22 [36] and, based on our observations, gives rise to O and T variants, suggesting that it has some level of variable capsule production. Of our phages, Maestro is considered a capsule targeting phage, specific to the capsule type KL116, with resistance to Maestro being associated with mutations in the capsular glycosyltransferase protein Gtr76 [20]. Interestingly, Maestro has previously been clinically used against *A. baumannii* strain TP1 (KL116 [20]) as part of a cocktail including several other phages that were initially isolated on AB5075 capsule negative mutants [13,20,33]. Its morphological and genetic similarities with AB5075 capsule negative targeting phages [20] therefore indicated to us that Maestro might also be able to infect AB5075 – but only the AV-T or capsule negative states. CO01 has also been described as a capsule (KL2) targeting phage of *A. baumannii* strain A9844, yet while capsule targeting it might preferentially target strains with capsule locus types producing less capsule than AB5075 and its VIR-O state (see [30,40,41] and figures therein). Little is known about phages FG03 and FG04 [33], but as FG03 could also infect 423159 we hypothesised it to have similar properties to Maestro and CO01. FG04 was included as a negative control in our experiments seeing as it does not plaque on MRSN strain 423159.

**Fig 1:**
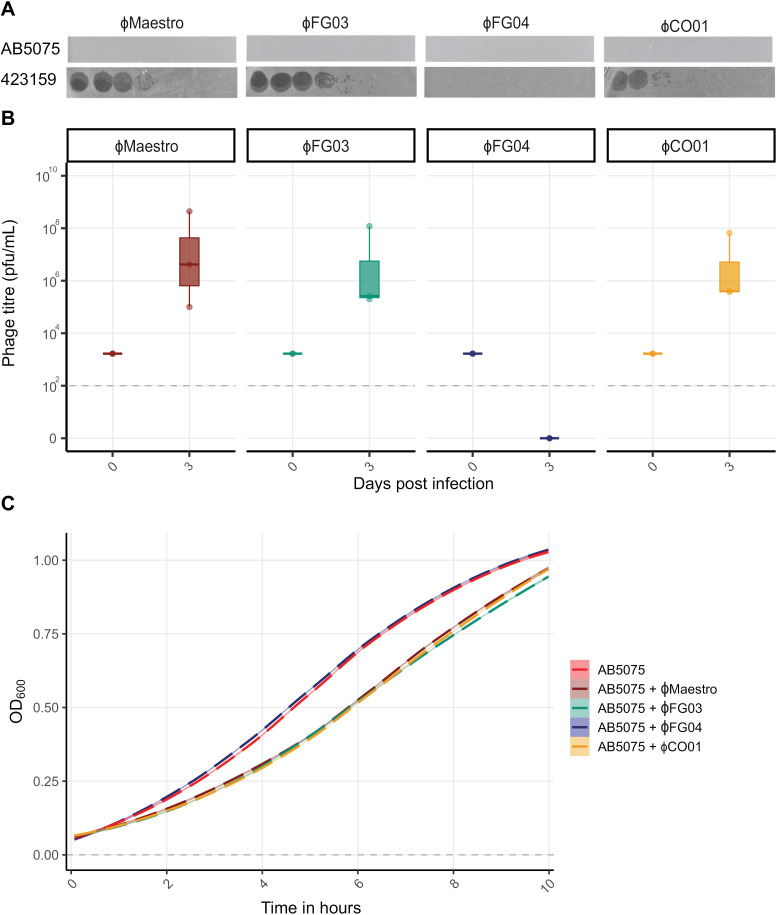
Some phages can infect in liquid culture but not on a bacterial lawn of the same host strain. **A** Bacterial lawns of AB5075 and 423159 with serial dilutions of phages Maestro, FG03, FG04, and CO01. None of these phages plaque on AB5075 but three do on 423159, which was used as an indicator for which phages might be able to infect AB5075 in liquid cultures. **B** In liquid culture, Maestro, FG03, and CO01 are all able to infect AB5075 (phage plaques were counted on lawns of 423159 as they do not plaque on AB5075 as shown in A). FG04 cannot infect AB5075 on a lawn or in liquid culture (*n* = 3 per phage). Horizontal dotted line indicates the limit of detection for our phage spot assays, where the phage is considered extinct. **C** OD_600_ growth assays (MOI = 1) show how phages Maestro, FG03, and CO01 are all somewhat able to limit initial AB5075 growth, whereas FG04 does not affect bacterial growth. All data are mean ± 95% confidence intervals.

Next, to test the ability of these other phages to infect AB5075, we inoculated them with AB5075 in liquid culture for three days with daily transfers into fresh medium. All plaque assays were performed on lawns of both 423159 and AB5075 at three days post infection, both to make plaque counting possible and to test if plaques would form on AB5075 after three days of evolution. Day three was used as the end point since any phage not actively amplifying would be diluted out of the population by that point due to daily 1:100 dilutions of both bacteria and phage into fresh growth medium. Doing this, we found that the phages Maestro, FG03, and CO01 all infect and amplify on AB5075 in liquid culture, yet none of them ever plaque on AB5075 (Fig 1B). Therefore, we used 423159 throughout all experiments to quantify plaque forming units (pfu) for all phages. The discrepancy in phage infectivity between culturing conditions was unexpected and something we observed throughout our work, warranting more extensive work that is beyond the scope of this paper. Our initial findings based on silica density gradients, however, indicated that AB5075 capsule production is on average lower when grown as a lawn on agar plates, as well as more heterogeneous (S1 Fig). The lower amounts of capsule on agar were not due to the VIR-O variant switching to the AV-T form. The mechanistic details of this effect are as of yet unknown, but might be due to mechanosensation [42].

Finally, we also tested the phages’ virulence potential against AB5075 using optical density measurements (OD_600_) (Fig 1C). To this end, the phages were added to bacterial cultures at a multiplicity of infection (MOI: ratio of phages to hosts) of 1 before taking readings every 5 minutes for 24 hours. This revealed how phages Maestro, FG03, and CO01 all somewhat reduced initial bacterial growth (Fig 1C: linear model with multiple comparisons of means; adjusted R^2^ = 0.98, F_14,3585_ = 1.5 x 10^4^, *p* < 0.001. AB5075 vs AB5075 + Maestro: *t* = -7.03, *p* < 0.001; AB5075 vs AB5075 + FG03: *t* = -6.47, *p* < 0.001; AB5075 vs AB5075 + CO01; *t* = -8.15, *p* < 0.001), whereas phage FG04 did no*t* affect AB5075 grow*t*h (Fig 1C: AB5075 vs AB5075 + FG04; *t* = -1.68, *p* = 0.45). We note that several of our phages caused aggrega*t*es to form, which is likely to have affected the OD_600_ readings (S2 Fig) and is why the endpoint for all growth rate experiments is at 10 hours to avoid our analyses being skewed by aggregate effects.

**Fig 2:**
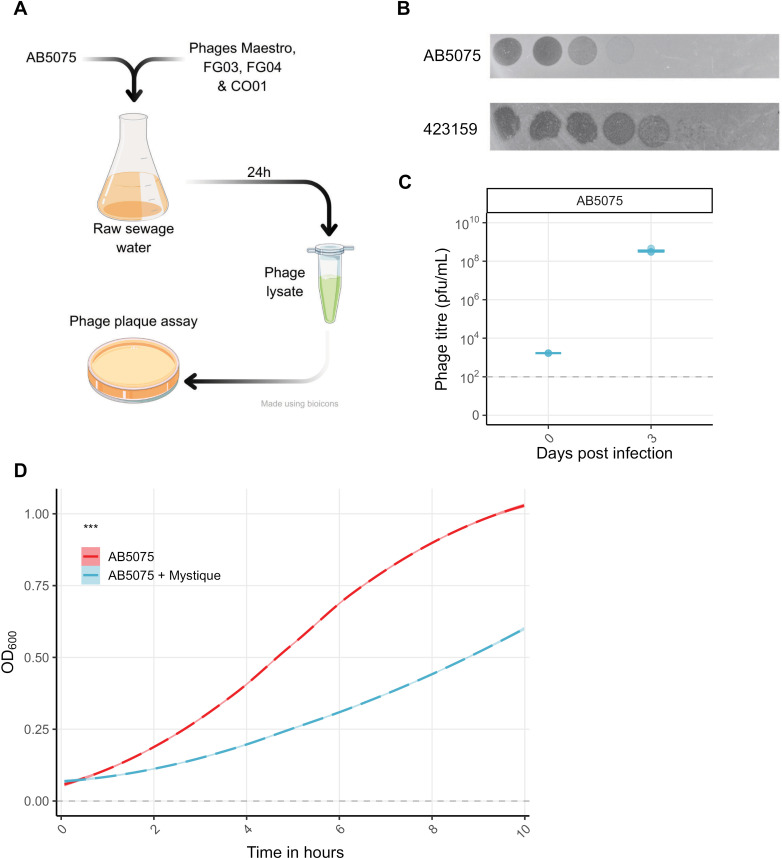
Mystique is a novel lytic phage targeting *A. baumannii* AB5075. **A** Illustration of the phage isolation process, made using clip-art from Bioicons (https://bioicons.com/) under license CC-BY 3.0 Unported. **B** Serial dilution of Mystique phage lysate after isolation and purification (always using AB5075 as host for phage amplification) to ensure the presence of only one phage, pipetted onto lawns of *A. baumannii* strains AB5075 and 423159. Horizontal dotted line indicates the limit of detection for our phage spot assays, under which phage is considered extinct. **C** Mystique phage titres after three days of co-inoculation and daily passaging with AB5075 (*n* = 3). Horizontal dotted line indicates the LB control. All data are mean ± 95% confidence intervals. **D** Growth curves using optical density at 600 nm (OD_600_) of wild-type AB5075 grown either alone or in the presence of phage Mystique (*n* = 6 per treatment; *** *p* < 0.001) at an MOI of 1.

Based on these findings, we hypothesised that Maestro, FG03, and CO01 could infect AB5075 when it expresses reduced capsule production, and we subsequently added these phages to the sewage before inoculation with AB5075, which resulted in the isolation of phage Mystique (Fig 2). FG04 showed no potential of being able to infect AB5075 (Fig 1), but was nonetheless included to reduce the small probability of drastic mutational changes to a potential phage surface receptor, as any such change could come at the cost of making the host susceptible to this other phage. By utilising these evolutionary trade-offs, the addition of these phages to the sewage filtrate may have aided in the isolation of phage Mystique by constraining resistance evolution while not confusing the results due to their inability to plaque on AB5075 – unlike Mystique. After our successful phage isolation, we discovered that Mystique can infect both the VIR-O and AV-T states of AB5075 (S3 Fig). The direct impact of our phage-cocktail approach to phage isolation is therefore uncertain but may still have constrained other means of host resistance evolution.

While Mystique does cause bacterial clearance on a lawn of AB5075, it does not form individual plaques, which made it difficult to verify the presence of a single phage (Fig 2B). Interestingly, when pipetting a serial dilution of the sewage lysate on various strains of *A. baumannii*, phage plaques were observed on *A. baumannii* 423159 (Fig 2B), from which an individual plaque was picked and purified three times in liquid cultures of AB5075 to ensure the isolation of one individual phage. Out of all strains tested (*n* = 103), individual phage plaques were only observed on 423159, hence its use throughout our study. Plaque assays on 423159 were consequently used to assess Mystique’s infectivity in liquid cultures of AB5075, which revealed the phage’s ability to infect, amplify, and remain in the population over the course of three days (Fig 2C). As a control, phage-free supernatant from AB5075 cultures was also spotted on lawns of 423159, which resulted in no plaques or zone of clearance. Finally, phage virulence assays using OD_600_ at an MOI of 1 showed that Mystique can limit growth of AB5075, even more so than phages Maestro, FG03, and CO01 (Figs 2D and 1C: linear model; adjusted R^2^ = 0.99, F_8,1431_ = 1.2 x 10^4^, *p* < 0.001. AB5075 vs AB5075 + Mystique; *t* = -7.77, *p* < 0.001). Again, we would here like *t*o point out that the presence of some phages, including Mystique, caused the formation of bacterial aggregates (S2 Fig), and so to avoid our analyses being skewed by aggregate effects all growth rate experiments have an endpoint at 10 hours post inoculation.

### Mystique sequencing, annotation, and assembly

**Fig 3:**
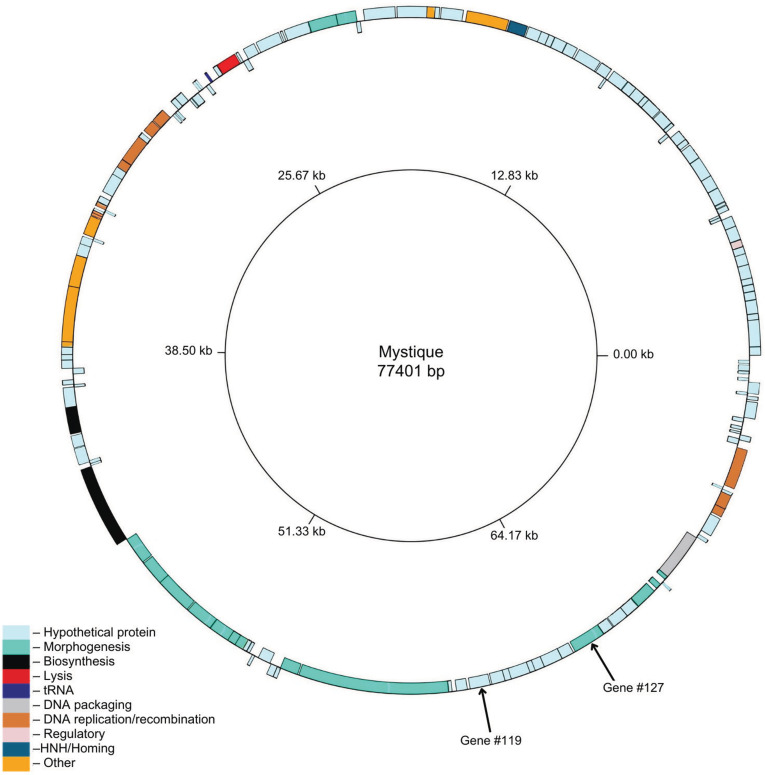
The annotated genome of Mystique. Mystique has a genome size of 77,401 bp, with 154 predicted genes out of which the majority are hypothetical and 38 have putative functions. Figure generated using GenomeVx [50].

After the isolation and purification of Mystique, DNA was extracted from the phage lysate followed by Illumina and Oxford Nanopore sequencing. Once the hybrid (long and short sequencing reads) assembly of the genome was complete, Mystique was found to be a dsDNA phage with a GC-content of 40%, with 154 predicted genes of which 115 are annotated as hypothetical proteins, while 38 have assigned putative functions (GenBank accession number PQ438181). Additionally, one tRNA gene was identified (Fig 3). Next, to control for Mystique potentially being an induced prophage, we used NCBI BLAST with the megablast search tool to compare Mystique’s genome with that of its isolation host (AB5075) and found no significant similarities. We further used the PhaTYP pipeline [43] to predict the phage’s lifestyle, which identified Mystique as a virulent phage (rather than temperate) with a probability score of 0.9998588.

Next, we looked for relatedness between Mystique and other known *Acinetobacter* phages, and found it to be similar to phage vB_AbaS_TCUP2199 (GenBank accession number ON323491.1 [44]) with 96.63% identity across 97% of the Mystique genome. Two phages are suggested to be considered the same species if their genomes are more than 95% identical across their full genome length [45]. So, while they are closely related, Mystique and vB_AbaS_TCUP2199 likely belong to the same genus but are not the same species. Another phage, EAb13 (GenBank accession number OQ717042.1 [46]) has 84.47% identity across 8% of the Mystique genome. No significant genetic similarities were found when comparing Mystique to phages Maestro and CO01, and to our knowledge, the sequences for FG03 and FG04 are not publicly available. Where possible we also compared phage morphologies, with Mystique being morphologically a siphovirus, unlike the other phages (S4 Fig). We were unable to compare Mystique’s morphology to that of FG04 as we did not find a strain this final phage could infect and amplify on before running out of sample. Finally, we used DefenseFinder [47–49] to look for the presence of potential defences and anti-defences, finding none from either category in the phage genome.

### CryoEM analysis reveals an icosahedral T=9 capsid and a long flexible tail

**Fig 4:**
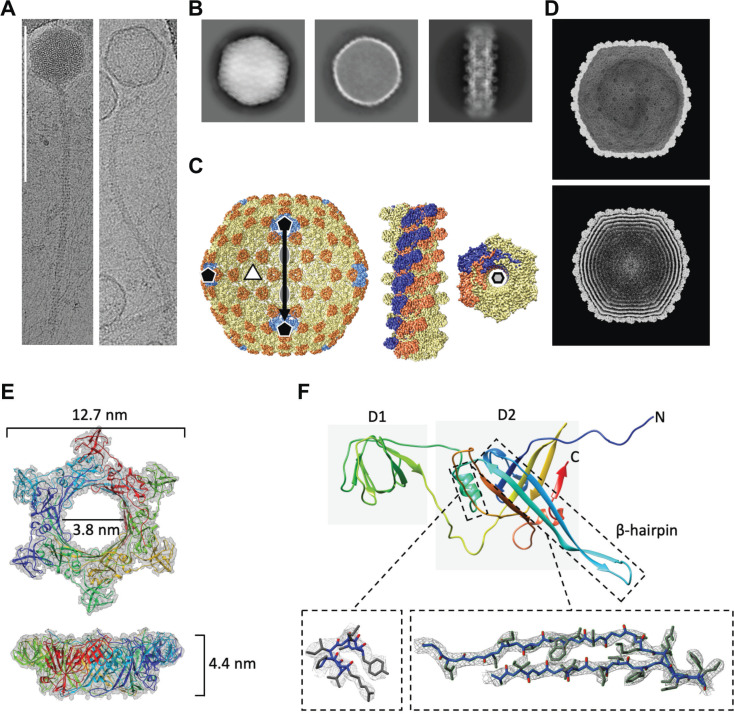
CryoEM reconstruction of bacteriophage Mystique and structural tail details showing hexameric assembly. **A** Individual phage particles from CryoEM micrographs showing bacteriophages with a full head (left) and an empty head (right). Scale bar 200 nm. **B** Selected 2D classes from data processing of phage particles (left = full head, middle = empty head, right = tail). **C** Left: 3D reconstruction (with icosahedral symmetry applied) of the Mystique head showing icosahedral symmetry axes. For this map, particles from empty (2,793) and full (3,207) heads were combined resulting in a 4.5 Å resolution reconstruction (blue = pentamers, yellow = hexamers, orange = decorating protein). Right: helical reconstruction of Mystique tail with C6 symmetry. Two adjacent individual helical strands are coloured in blue and orange. **D** Independent CryoEM reconstructions of empty (top) and full (bottom) capsids displayed at high thresholds. **E** Helical asymmetric unit of Mystique’s tail unit with a 6-fold symmetry. The final refinement had a helical twist of 17.9 degrees and a helical rise of 42 Å. **F** Tail protein monomer with two main domains and a β-hairpin. Boxes show the alpha helix and a β-hairpin fitted into a 3.2 Å map.

To gain insights into the Mystique morphology and structure, we analysed the phage lysate with negative-stain transmission electron microscopy (TEM) followed by cryo-electron microscopy (cryoEM). Negative staining was done to verify sample viability and concentration, after which the phage was further concentrated using polyethylene glycol (PEG) precipitation [51]. CryoEM data acquisition revealed that phage particles were present in vitreous ice on cryoEM grids, yet most of the tails were detached from the heads, possibly due to the harsh conditions of PEG precipitation [52]. In total, 7200 head particles and 363,000 tail segments were picked from micrographs for further analysis. Interestingly, the initial 2D classification revealed that the Mystique head particles were in two distinct states – one empty and one full (Fig 4A and 4B). A total of 6000 head particles and 191,150 tail segments were used for the final refinement, producing maps at 4.5 Å and 3.2 Å resolution respectively (Fig 4C). Additionally, independent 3D reconstruction of particles from both empty and full states (Fig 4B) produced lower resolution but identical maps, suggesting that phage capsid structure does not depend on the presence or absence of nucleic acids (Fig 4D).

Through cryoEM, we further found that Mystique’s head has an icosahedral T=9 symmetry (h=3, k=0 [53]), and AlphaFold2 structure prediction of the protein encoded by Mystique gene number 127 (Fig 3) revealed an HK97 fold [54], suggesting a similar organisation to that of other phages with HK97 capsids (S5A Fig). However, the resolution of the phage heads was not sufficient to unambiguously trace a backbone model in the cryoEM map density. Instead, we used rigid-body fitting to manually fit the predicted model into the map, assembling an asymmetric unit (S5B Fig) before applying icosahedral symmetry to produce a closed cage that matched the EM density (S5C Fig). Unfilled densities may belong to a yet unidentified “cement” or “decoration” protein common for bacteriophages with HK97-like folds [54].

Next, the cryoEM map of the phage tail was used for protein sequence prediction through *de novo* modelling using ModelAngelo [55]. The resulting model was then used to search through Mystique’s genome, identifying gene number 119 as the tail protein (Fig 3). The protein sequence derived from gene number 119 was further used to build a model which was refined with C6 symmetry (Fig 4E) before being used as input for Foldseek [56]. This revealed that YSD1, a phage infecting *Salmonella* [57], has a highly homologous tail structure, despite low sequence similarity (S6A and S6B Fig). The Mystique tail monomer is organised into two major domains: the external D1 domain and a core D2 domain. A β-hairpin of each subunit in a hexamer interacts with a preceding and two subsequent subunits, thus forming a highly interlocked assembly (Fig 4F). A third domain (D3) is absent in the Mystique tail, but present in the YSD1 tail [57], although poorly resolved. Similar to YSD1, Mystique’s tail also has a highly negatively charged lumen necessary for the translocation of nucleic acid from the head to the host (S6C Fig). Overall, cryoEM analysis was key in identifying Mystique structural proteins, particularly the tail protein, and highlights how structural homologs do not always correspond to genetic relatedness.

### Mystique is broad host range *Acinetobacter* phage

With Mystique sequenced and structurally characterised, we next set out to determine its more exhaustive *A. baumannii* and *Acinetobacter* host range. Most *A. baumannii* phages have narrow host ranges restricted by capsule/K locus type [32,33,58], and we hypothesised the same to be true for Mystique. However, Mystique can infect at least two different capsule loci (AB5075 is KL25 [26], while 423159 is KL22 [36]). Structurally, KL22 strains produce either K3 or K3-v1 capsular polysaccharide [59], which share no clear structural similarity in either sugar content or linkages to the K25 polysaccharide [59,26]. Based on this dissimilarity, we expected Mystique might be able to infect multiple capsule types, in addition to KL25 and KL22. To test this, we conducted Mystique plaque assays on 103 highly diverse clinical isolates of *A. baumannii*, 100 of which were from the MRSN diversity panel [36] as well as FZ21 [17] and TP1 [13], with AB5075 included as a positive control. The MRSN diversity panel in particular is meant to represent the genetic diversity of *A. baumannii* as a species [36].

Based on these plaque assays, we found that Mystique can infect at least 88 of the 103 strains tested (85.4%) (S1 Data). However, because we previously showed that the environment for assessing phage infectivity matters (Fig 2), we hypothesised that Mystique might have an even broader host range than indicated by plaque assays if tested in a liquid culture rather than on a bacterial lawn. To determine this, we next inoculated Mystique in broth culture with the 15 strains it does not plaque on, with daily transfers for three days before phage titres were determined by plaque assays on 423159. In liquid culture, in addition to the 88 already confirmed, Mystique could also infect MRSN strains 334, 1171, 7153, 11816, 22112, and 337038 (S7 Fig) for a total of 94 out of the 103 strains tested (91.3%). These results further highlight the importance of testing phages in liquid culture, as there was once more a large discrepancy in the observed results between environments (lawn vs liquid) used to test for phage infectivity (S7 Fig).

**Fig 5:**
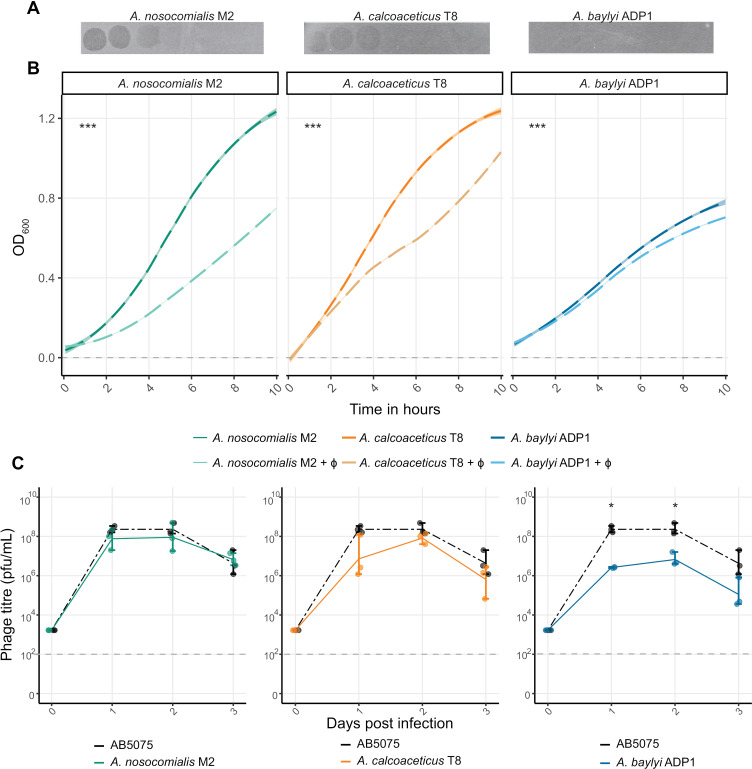
Mystique can infect other species of *Acinetobacter.* **A** Serial dilution of phage Mystique spotted on lawns of *A. nosocomialis* M2, *A. calcoaceticus* T8, and *A. baylyi* ADP1 giving initial indication of this phage’s ability to infect all three, although barely so in the case of *A. baylyi*. **B** Growth assays using OD_600_ (MOI = 1; *n* = 6) further supporting the notion of Mystique being a multi-species phage, at least for *A. nosocomialis* and *A. calcoaceticus*, and **C** liquid culture infection over three days confirmed the phage’s ability to infect, amplify and persist in cultures of all three species of *Acinetobacter.* Horizontal dotted line indicates the limit of detection for our phage spot assays, under which phage is considered extinct (*n* = 3; linear model with multiple comparisons of means: * *p* < 0.05). All data are mean ± 95% confidence intervals.

Knowing Mystique has a remarkably broad *A. baumannii* host range, we then set out to test its ability to infect other *Acinetobacter* species. Using plaque assays, phage virulence assays using OD_600,_ and liquid culture assays as described for other experiments above, we discovered how Mystique can also infect and amplify on *Acinetobacter nosocomialis* M2, *Acinetobacter calcoaceticus* T8, and *Acinetobacter baylyi* ADP1 (Fig 5). While barely visible on lawns of *A. baylyi*, Mystique did have various levels of virulence capacity against all three species, although to a much lesser extent against *A. baylyi* (Fig 5B: linear model with multiple comparisons of means; adjusted R^2^ = 0.97, F_16,4303_ = 9957, *p* < 0.001. *A. nosocomialis* no phage vs phage: *t* = 104.7, *p* < 0.001; *A. calcoaceticus* no phage vs phage: *t* = 70.42, *p* < 0.001; *A. baylyi* no phage vs phage; *t* = 13.29, *p* < 0.001). Addi*t*ionally, Mystique can amplify and persis*t* in *A. baylyi* liquid culture, implying infectivity but to a more limited extent (Fig 5C). Whether Mystique’s limited ability to affect *A. baylyi* is a phage- or host-specific effect remains to be determined, as is Mystique’s potentially more extensive *Acinetobacter* host range.

**Fig 6:**
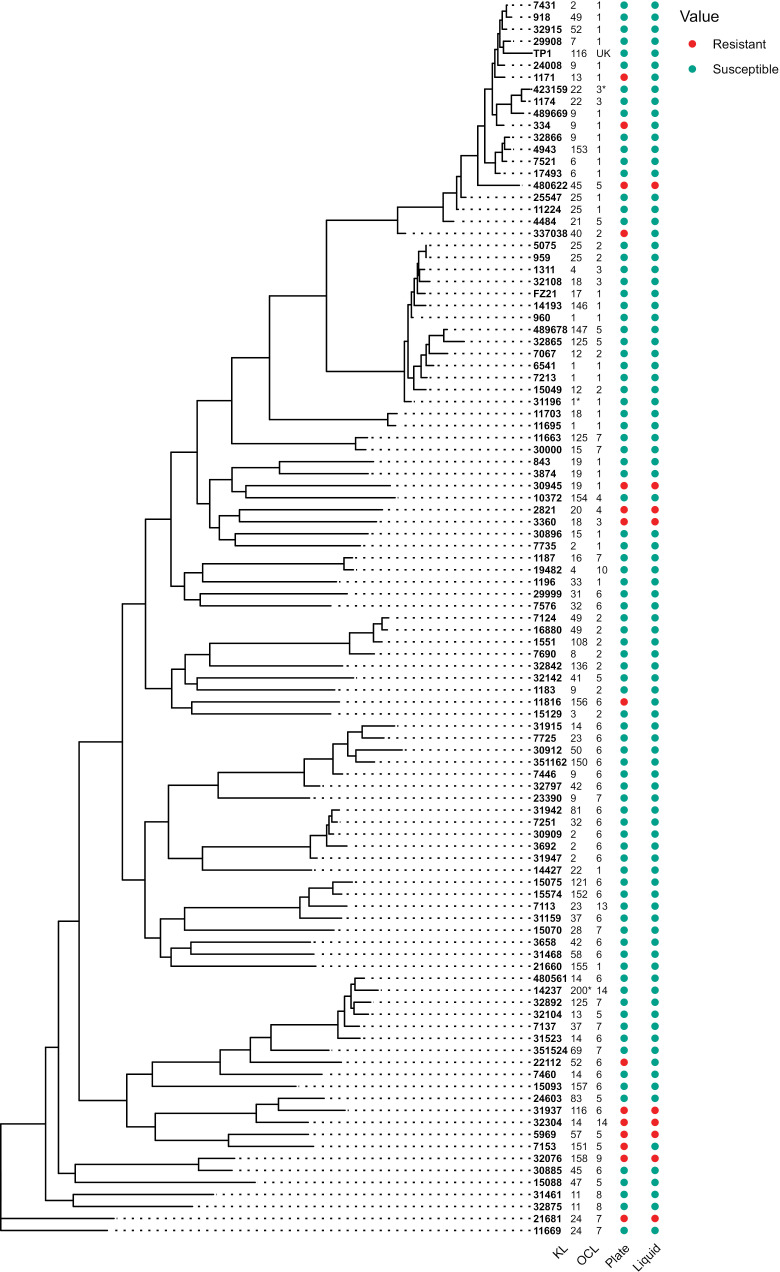
Phylogenetic tree of strains that Mystique was tested on. Phylogenetic tree of *A. baumannii* stains that are susceptible or resistant to phage Mystique after having been tested using both plaque (labelled as ‘plate’) and liquid assays (labelled as ‘liquid’). KL indicates the strain’s capsule type, while OCL is the lipooligosaccharide outer core locus (UK = unknown, * = likely variant type), and were either acquired from the literature or through using Kaptive [36,61,62].

We also made a phylogenetic tree and tested for a potential phylogenetic signal across our *A. baumannii* strains by first assessing strains susceptible based on plaque assays (Fig 6). This gave us a D value of 0.3 (*p* = 0), which is a measure of phylogenetic signal for a binary trait, where a number closer to 1 indicates a trait evolved from random motion [60]. With susceptibility in liquid as the binary trait on the other hand, the D value decreased to 0.084 (*p* = 0.02), strongly indicating that resistance to Mystique is a non-random and heritable trait (S8 Fig). Despite the host range for most other *A. baumannii* phages being restricted by capsule type/K locus type [32,33,58], there were no clear similarities between the susceptible strains’ K locus type (KL) or lipooligosaccharide outer core locus (OCL) that could answer why a specific strain is resistant or susceptible to Mystique (Fig 6).

### The capsule synthesis pathway plays a key role in Mystique infection

Following the host range assays, we set out to try and determine the Mystique phage receptor. Due to its broad host range, we anticipated that the receptor must be something all strains have in common. This, combined with the knowledge that most other *A. baumannii* phages use the capsule as a receptor [27,32,33,58], we hypothesised that the bacterial capsule functions as the Mystique phage receptor.

**Fig 7:**
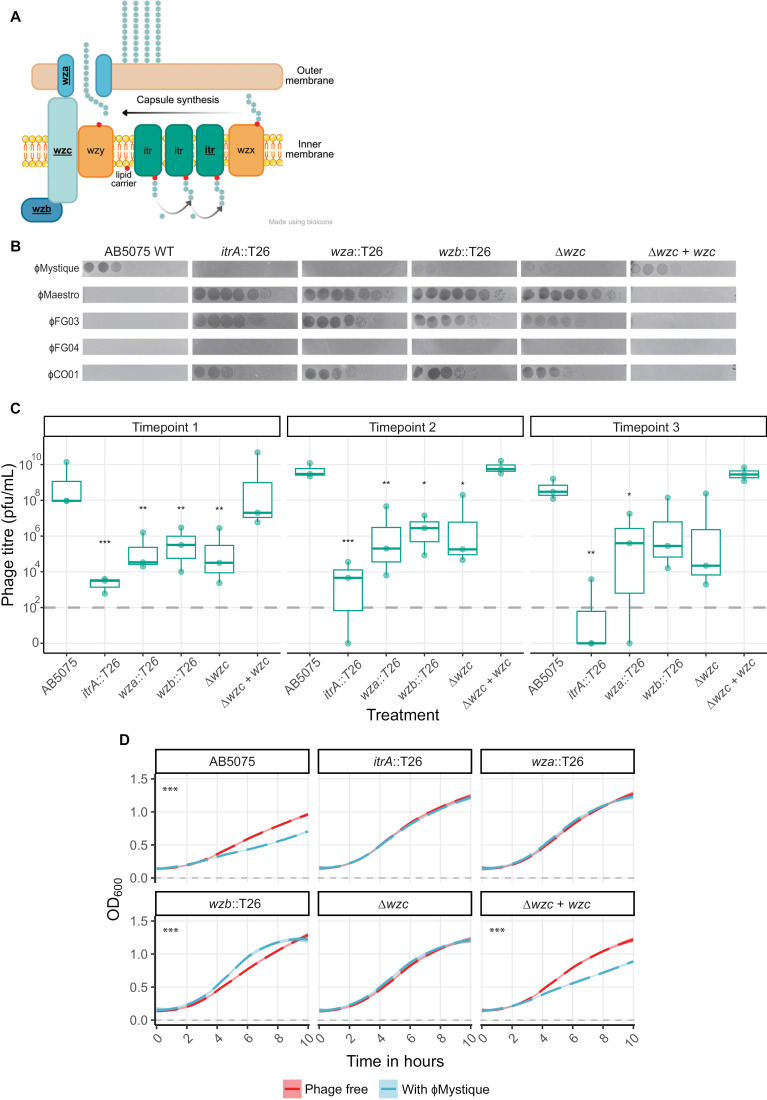
The capsule plays an important role in AB5075 susceptibility to Mystique, and susceptibility is dependent on culturing conditions. **A** The *A. baumannii* capsule synthesis pathway, adapted from Singh *et al*. 2019 [63], made using clip-art from Bioicons (https://bioicons.com/) under license CC-BY 3.0 Unported. **B** Serial dilution plaque assays of phages Mystique, Maestro, FG03, FG04, and CO01 on the AB5075 wild-type, *itrA*::T26, *wza*::T26, *wzb*::T26, ∆*wzc*, and ∆wzc + *wzc*. **C** Mystique phage titres over time in liquid broth culture after inoculation with the AB5075 WT or its capsule mutants. Horizontal dotted line indicates the limit of detection for phage spot assays, under which phage is considered extinct. **D** Growth curves using optical density at 600 nm (OD_600_) of wild-type AB5075 and its capsule mutants as listed above, grown either in the presence or absence of phage Mystique (*n* = 3 per treatment) at an MOI of 1. Horizontal dotted line indicates the LB control. All data are mean ± 95% confidence intervals (*n* = 3; linear model with Tukey post hoc testing for multiple comparisons: * p < 0.05, ** p < 0.01, *** p < 0.001).

To test this hypothesis, we used mutants of various capsule synthesis genes, specifically the *itrA*, *wza*, *wzb*, and *wzc* genes that encode important components of the capsular polysaccharide synthesis pathway [63] (Fig 7A). For example, ItrA is the initiating transferase, which is required for both capsule synthesis and protein O-glycosylation, while Wza, Wzb, and Wzc together form a complex that coordinates the assembly and export of the capsular polysaccharide [32,63,64] (Fig 7A). Disrupting these genes results in near complete or complete capsule loss [40,65,66], and reduced efficacy of plaquing would indicate that the capsule does play a role during phage infection.

Plaque assays revealed that for all four mutants there was a drastic decrease in phage Mystique infectivity (Fig 7B). Specifically, disruption of the *itrA* and *wza* genes seemed to confer complete phage resistance while we still observed some clearance on the *wzb* and *wzc* mutants, implying partial resistance (Fig 7B). We also performed plaque assays on a complemented strain: AB5075∆*wzc* + *wzc*. Making the *wzc* gene functional again fully reversed the previously observed phage resistance, resulting in it becoming re-susceptible at the same level as the wild-type AB5075 (Fig 7B). Additionally, we tested the other phages used during phage isolation on the same capsule mutants and found an inverse pattern where the phages that can amplify in liquid but not on a bacterial lawn of AB5075 (Fig 1) do cause lysis on all AB5075 capsule mutants (Fig 7B).

Next, we tested how well Mystique would amplify on these mutants in liquid culture, following our observations on the importance of environmental conditions when assessing phage susceptibility/resistance (Figs 2B, 4 and S1). Doing this (with daily 1:100 dilutions into fresh media) revealed that Mystique can amplify in the population when inoculated with all capsule mutants, but that this was to some degree mutant dependent (Fig 7C). In particular, we observed two cases of phage extinction in the presence of the *itrA*::T26 mutant as well as drastically limited phage amplification, indicating this gene as being especially important in host resistance to phage Mystique. Additionally, while all mutants negatively affected Mystique amplification on day 1 and 2, by timepoint 3 there was only a significant effect of treatment (wild-type or isogenic mutant) on phage titre for the *itrA*::T26 and *wza*::T26 mutants, with consistently lower levels of phage for the duration of the experiment (with the *itrA* mutant being consistently close to the detection threshold of 10^2^ pfu/mL). This is consistent with these two mutants also having the strongest negative effect on phage infectivity on a bacterial lawn. It is here worth pointing out that the ability of a phage to persist and even somewhat amplify on highly resistant bacteria can facilitate the evolution of escape phages (phages with mutational changes that overcome host resistance) to emerge over time [67], which could be why we saw one case of phage persistence with the *itrA* mutant.

We also measured bacterial growth in the presence and absence of phage Mystique for all mutants, measuring OD_600_ over 10 hours after co-inoculation with the phage at an MOI of 1. These measurements revealed how Mystique only has a negative impact on bacterial growth for the wild-type and the complement strain AB5075∆*wzc* + *wzc* (Fig 7D: linear model with multiple comparisons of means; adjusted R^2^ = 0.85, F_25,21130_ = 4758, *p* < 0.001. AB5075 without or with phage: *t* = 6.15, *p* < 0.001; ∆*wzc* + *wzc* wi*t*hout or with phage: *t* = 8.98, *p* < 0.001). Sta*t*istically, the *wzb*::T26 mutant seemed to be performing better in the presence of the phage (t = -5.5, p < 0.001), however, this could be due to natural variation and some other, not phage-specific, effect.

Finally, we looked for mutational patterns in these same genes across all strains Mystique was tested on, comparing them to the AB5075 WT, but found no clear pattern in this initial analysis that could point to one particular gene or mutation that would provide Mystique resistance (S9 Fig). Further, we did not manage to isolate a spontaneous mutant of AB5075 that was resistant to Mystique at a satisfactory level, as Mystique was able to cause various levels of clearance on lawns for all isolated clones (*n* = 35).

## Discussion

Here, we report on the isolation and characterisation of Mystique, a novel lytic *Acinetobacter* phage, as well as underscoring the limitations of conventional phage techniques. There was previously no well-characterised or readily available phage against the important clinical model strain AB5075, and Mystique was isolated to bridge this gap, providing both researchers and clinicians with a potential phage for further studying *Acinetobacter* – phage dynamics. Mystique is a double-stranded DNA phage with the morphology of a siphovirus, a T=9 icosahedral head and helical C6 symmetric tail structure. While genetically similar to other *A. baumannii* phages, cryoEM revealed that Mystique was more structurally similar to phages such as YSD1 that infects *Salmonella* [57], highlighting the importance of studying structure in combination with genetics, as this might tell us more about phage biology than when considering either factor in isolation.

In addition to targeting AB5075, Mystique has a remarkably broad host range, being able to infect 88 (85.4%) of 103 highly diverse *A. baumannii* strains based on plaque assays. During the phage isolation process, however, we also consistently observed how some phages are able to infect AB5075 in liquid culture while simultaneously not plaquing on bacterial lawns (Fig 1), the latter of which is the standard method for isolating phages and testing infectivity [38]. When also assessing Mystique in liquid culture, its final host range was at 94 (91.3%) out of the 103 strains tested. Additionally, and remarkably so, we found that Mystique is not restricted to strains of *A. baumannii*, being able to infect other species of *Acinetobacter* as well. Specifically, we found that Mystique can infect more closely related species, such as *A. nosocomialis* and *A. calcoaceticus*, and distantly related species like *A. baylyi* – although to a lesser degree (Fig 5).

Mystique’s broad host range is to-date unique, as most other *A. baumannii* phages are considered to have narrow host ranges restricted by capsule type [32,33,58]. Mystique infectivity, on the other hand, is not restricted by capsule type, and so its broad host range is quite likely due to multiple complex factors. For example, it is possible that Mystique has multiple phage receptors. Based on our findings however, we can say that the phage receptor is 1) a structure shared across multiple *Acinetobacter* species and is 2) linked to capsule synthesis and/or glycosylation. For instance, our results revealed how several AB5075 capsule mutants conferred drastically reduced infectivity compared to the wild-type using plaque assays (Fig 6B). Making the *itrA* gene non-functional resulted in the strongest reduction in infectivity, with two out of three populations showing phage extinction by timepoint 3 as well as this mutant exhibiting unimpaired bacterial growth when in the presence of phage (Fig 6C and 6D). Mystique being able to still infect some of these mutants in liquid culture might in part be explained by how capsule production is reduced when grown on a lawn on agar plates compared to liquid (S1 Fig). It may also be that mutations that alter the latter stages of capsule synthesis (*wza*, *wzb*, and *wzc*) are still permissive to infection. These might have some level of surface capsule that, although missing key sugar residues, is still enough to allow for Mystique to infect the cell.

Further, the disconnect between the standard assays on bacterial lawn and liquid culture that we continuously observed throughout our study likely means we are missing a fundamental property when it comes to phage-bacteria dynamics. Particularly when it comes to *A. baumannii* and potentially also other bacteria with similar ability to regulate capsule thickness. For instance, *E. coli* was recently found to regulate capsule thickness and consequent masking of the phage receptor in response to cell surface pressure (mechanosensation) with downstream effects on phage susceptibility [42]. This effect was lost if using an *E. coli* ∆*wza* mutant [42], which we note is one of the same genes disrupted in our work. It is therefore not unlikely that a similar effect may be involved for *A. baumannii*, where mechanosensation results in regulatory expression changes modifying capsule thickness, although we observed reduced and more heterogeneous capsule production for bacteria grown on a lawn (S1 Fig). Similar discrepancies between lawn and liquid assays have previously been reported for *Salmonella*, where plaque assays on a bacterial lawn indicated greater phage sensitivity than liquid cultures [68] – the inverse of our findings for *A. baumannii*. It is therefore clear that this is an avenue for further study, and more work is needed to elucidate what causes some phages to be unable to infect on bacterial lawns but not in liquid environments.

This imperfect mapping between testing environments further highlights the complex nature of bacteria-phage dynamics and the need for research on the finer mechanistic details at play when phages use the *A. baumannii* capsule as their receptor. This was made clear by our results showing how multiple phages in our collection that use the capsule for their focal strains as the phage receptor [20,33] were also only able to lyse AB5075 capsule negative mutants. Further, research on bacteria-phage dynamics for *Bacteroides intestinalis* has previously shown how phase variation of individual capsular polysaccharides is an important mechanism for bacteria and phages co-existence, allowing for bacteria and phages to multiply in parallel [39]. This might also be true for *A. baumannii*, and may in part be why we see bacteria and phage persist together over the course of three days. This effect is again also likely to be affected by culturing conditions, which will influence the regulation of capsule production (S1 Fig) in ways that may facilitate or limit phage infectivity in ways that are likely strain- and/or species-dependent. Overall, this indicates that we are still missing crucial pieces of the puzzle regarding how various phages interact with a diverse and plastic surface structure like the *A. baumannii* capsule [29,30,63].

In conclusion, Mystique is a novel phage capable of infecting a wide range of *A. baumannii* strains and other *Acinetobacter* species. Additionally, the Mystique phage isolation, structural analysis, and characterisation process highlights the importance of re-evaluating traditional phage isolation techniques and adopting a multifaceted approach to phage research. By interrogating the interplay between phages and their bacterial hosts in diverse environmental contexts, we can gain deeper insights into the mechanisms of phage resistance in ways that will aid us in devising more robust strategies for phage therapy against *A. baumannii* and other bacteria with complex capsules.

## Materials and Methods

### Bacterial strains and phages

The strain used for isolating phage was *A. baumannii* AB5075_UW [25]. An additional 102 *A. baumannii* strains were used to assess phage host range. These included 100 diverse clinical isolates from the Multidrug-Resistant Organism Repository and Surveillance Network (MRSN) [34], clinical isolate FZ21 from Queen Astrid Military Hospital, Belgium [17], and clinical isolate TP1 from UC San Diego, USA [13]. The capsule mutants *wza*::T26, *∆wzb*:T26, and ∆*itrA*::T26) were obtained from the AB5075 transposon mutant library [69] while the *∆wzc* mutant and complemented ∆*wzc* + *wzc* are both previously described [66]. The additional phages used for this study were Maestro [20], FG03, FG04, and CO01 [33]. Three other species of *Acinetobacter* were also used for this study, represented by strains *A. nosocomialis* M2, *A. calcoaceticus* T8, and *A. baylyi* ADP1.

### Phage isolation

Mystique was isolated from raw sewage water from the R.L. Sutton Water Reclamation Facility in Atlanta, USA. Methods for isolation were adapted from previously used methods [27]. In brief, for a final concentration of 3 g of powdered LB medium (VWR) was mixed with 100 mL of raw sewage water before 100 µL of AB5075 was added. Bacteria were grown to exponential phase before being added to the sewage, after which they were incubated in the sewage mixture overnight at 37˚C at 180rpm.

After inoculation overnight, 1 mL of the sewage/LB mixture was sampled and centrifuged for 5 minutes at 8000 x g before the supernatant was filtered through a 0.22µM spin-X centrifuge tube filter (Corning) at 6000 x g to remove any remaining bacterial cells. 10 µL of this filtrate was added to 100 µL of AB5075 in exponential growth phase before incubation for 20 minutes at 37˚C and 180 rpm. After this second round of inoculation, the 100 µL mixture was combined with 2.5 mL of top agar (0.5% LB agar, VWR) before being poured over LB agar plates and placed in an incubator at 37˚C overnight. This, however, yielded no phage plaques and so 100 µL of supernatant from the raw sewage water/LB powder mixture was added to 6 mL of LB medium with AB5075 at exponential growth. In addition to the sewage filtrate, other known *A. baumannii* phages were added to the mixture in an attempt at limiting the rapid evolution of phage resistance overnight. These phages were Maestro [20], FG03, FG04, and CO01 [33]. These cultures were subsequently grown overnight, before taking 1mL of the culture to be centrifuged at 8000 x g for 5 minutes and filtering the resulting supernatant through a 0.22 µM filter. 5 µL of the filtrate was then pipetted on top of a lawn of AB5075 before incubation at 37˚C overnight. This resulted in bacterial clearance, and a 1-10 µL pipette tip was used to transfer a small amount from the centre of the zone of clearance into a fresh bacterial culture of AB5075. This was done three times, but no individual phage plaques were seen on AB5075.

To ensure the isolate only contained one phage, the lysate, after three days of passaging and purification, was also tested on an *A. baumannii* host for some of the other phages initially added in the cocktail: MRSN strain 423159 [36]. Clear individual phage plaques were observed on 423159 from which one was picked and purified three times (repeated plaque assays on 423159 followed by inoculation with AB5075).

### Phage virulence assays

For all optical density measurements (OD_600_), bacteria from overnight cultures were either grown alone or with phages from pure lysates mixed at an MOI of 1 (inoculum of ca. 10^7^ colony-forming units (cfu) per mL) in LB medium. Growth was measured over 24 hours, while shaking at 37˚C.

### Phage sequencing, annotation, and assembly

Phage DNA was extracted following already established methods for extracting phage DNA [70]. In short: 500µL of filter-sterilised phage lysate was incubated statically with 50 µL DNase I 10x buffer, 1 µL DNase I (1 U/µL), and 1 µL RNase A (10 mg/mL) for 1.5 h at 37 °C. Following this step, 0.5 M EDTA was added for a final concentration of 20 mM before 1.25 µL of Proteinase K was added after which the sample was inoculated at 56˚C for another 1.5 h. After this second incubation step, DNA was extracted following the instructions in the DNeasy Blood and Tissue Kit (Qiagen).

Following extraction, DNA fragmentation was performed using the NEBNext Ultra II FS DNA Library Prep Kit (New England Biolabs), an enzymatic fragmentation assay with an average fragment size of 380 bp. After fragmentation, the fragmented DNA was end-repaired, A-tailed, and ligated with Illumina-compatible adaptors using the same NEBNext kit. The ligated products were then amplified via PCR to enrich the library. The amplified libraries were purified using AMPure XP beads (Beckman Coulter) to remove any unbound adaptors and smaller fragments.

The prepared libraries were evaluated for size distribution and concentration using the Agilent 2100 Bioanalyzer (Agilent Technologies) with a High Sensitivity DNA Kit. Libraries exhibiting the desired size range and absence of primer-primer dimers were selected for sequencing on the Illumina NovaSeq 5000 platform, employing a paired-end 150 bp (PE150) read configuration to generate high-quality short reads.

For long-read sequencing, the extracted DNA was prepared for sequencing using the Ligation Sequencing Kit (SQK-LSK109) from Oxford Nanopore Technologies (ONT, Oxford, UK). The extracted DNA was quantified using a Qubit 4 Fluorometer (Thermo Fisher Scientific) to ensure an adequate amount of input material. The DNA was subjected to end-repair and dA-tailing using the NEBNext Ultra II End Repair/dA-Tailing Module (New England Biolabs). Following end-repair and dA-tailing, ONT’s proprietary sequencing adaptors were ligated to the DNA fragments using the Blunt/TA Ligase Master Mix (New England Biolabs) provided in the Ligation Sequencing Kit. The ligation reaction mixture was purified using AMPure XP beads (Beckman Coulter) to remove unligated adaptors and small DNA fragments, ensuring that only high-molecular-weight DNA with ligated adaptors proceeded to sequencing. The purified library was quantified again using the Qubit 4 Fluorometer to confirm the concentration and ensure that an adequate amount of library was available for sequencing. The prepared library was loaded onto a Flow Cell (R9.4.1) and sequenced on the Oxford Nanopore MinION device. Sequencing was performed according to the manufacturer’s standard operating procedures, and run conditions were monitored using ONT’s MinKNOW software. Sequencing continued until sufficient data was generated to achieve the desired genome coverage.

The sequencing data from both platforms were processed and analysed using standard bioinformatics pipelines. Short reads from the Illumina platform were trimmed and assembled using SPAdes, while long reads from the Nanopore platform were basecalled using Guppy and assembled using Canu.

Following sequencing, hybrid genome assembly and annotation were conducted on the Galaxy [71] and Web Apollo [72] phage annotation platforms. Unless otherwise noted, default parameters were used for all software. Long reads under 1kb were filtered out using Filtlong v.0.1.2 [73] and were subsequently quality checked using Nanoplot v.1.41.0 [74,75]. Flye v.2.9.1 [76,77] was used with –nano-hq and metagenomic assembly parameters [78] to obtain a consensus draft assembly. One circular contig 77,172 bp in length with 3,257x coverage was obtained. Short sequencing reads were rarefied to 100x coverage to improve assembly quality using FastQ Subset v.1.1 [79,80] and trimmed using the Trim Sequences tool v.1.0.2 [81]. Short reads were quality checked using FastQC v.0.72 [82] and aligned with the long read draft assembly using the Map with BWA-MEM tool v.0.7.17.2 [83,84]. That output was then used with pilon v.1.20.1 [85] to create a consensus hybrid assembly. The complete assembled contig was 77,401 bp long and was reopened using Reopen Fasta Sequences v.2.0 [80] in order to avoid interrupting genes. BLASTn [86] was used to find similarity to previously-identified phages.

The final Mystique assembly was imported into Apollo using the Galaxy Structural Phage Annotation Workflow v.2023.01 and the locations of genes were predicted as described in Ramsey *et al*. [79] using GLIMMER3 v.0.2 [87], MetaGeneAnnotator v.1.0.0 [88], and SixPack v.5.0.0 [89]. The criteria weighed in order to manually make final gene calls were assessment of gaps and overlaps between genes, the presence of a valid Shine-Dalgarno sequence, and the presence of a valid start codon. The presence of tRNAs was assessed using tRNAScan-SE v.0.4 [90] and ARAGORN v.0.6 [91]. When structural annotation was complete, functional annotation was conducted using the Galaxy Functional Phage Annotation Workflow v.2023.01 [79]. BLASTp [92,93] results were compared from the canonical phages, nonredundant-all phages, and Swiss-Prot databases to manually annotate putative functions. The geecee tool v.5.0.0 [94] was used to determine the GC content of the Mystique genome.

### Transmission electron microscopy and cryogenic electron microscopy

The Mystique phage sample was prepared using PEG precipitation [51] before being added to a plasma cleaned continuous carbon grid (30s, hydrogen, oxygen) and stained with 2% uranyl formate. The grids were imaged on a Hitachi 7800 TEM operated at 100kV, and data were collected at a pixel size of 1.77Å with a TVIPS XF416 (Gatan). Alternatively, PEG-precipitated samples were negatively stained with 1% uranyl acetate and imaged with a JEOL JEM-1400 TEM operated at 120kV and equipped with a Gatan OneView camera.

The phage sample was further concentrated 50-fold using Amicon Ultra 100k concentrators (100k MWCO). Grids were frozen on Quantifoil R2/2 Cu 300 mesh grids using a Vitrobot Mark IV (ThermoFisher Scientific) at 20°C and 100% humidity with a wait time of 0s, a blot time of 6.5s and a blot force of 1. The grids were then clipped into Autogrids and imaged on a Titan Krios G2 (ThermoFisher Scientific) equipped with a Gatan K3 direct electron detector and BioQuantum energy filter set to 20 eV slit width. Data were collected at a pixel size of 1.083Å, a dose of 49.90 e^-^/Å^2^, and a nominal defocus range of -1 to -2 µm. With fringe-free imaging (FFI), we were able to collect 6 images per hole, totalling in 8565 images for the data collection. Data were acquired using Leginon [95,96] on NYSBC Krios1, dataset m23oct30a.

All data were processed in CryoSPARC v4 [97] using standard workflow starting with raw frames. Frames were imported and motion-corrected using the patch motion job, and CTF was estimated using the patch CTF job. Micrographs were sorted to exclude any with a CTF estimation >4.6 Å. For both heads and tails, particles were first manually picked, then initial 2D classification was used to generate templates. For heads, template particle picking was used, resulting in ~26,000 particles picked that were triaged by 2D classification to yield a subset of 6000 particles. Initial models were generated using *ab-initio* jobs and resulting models were used for homogeneous refinement with icosahedral symmetry applied. For tails, we used a filament tracer and initially extracted ~1 million segments that were triaged by 2D classification to 191,000 particles. To generate an initial model, first an ab-initio job was used, followed by homogeneous refinement. The resulting map was used to identify initial helical parameters and was also used as an initial model for helical refinement. After initial helical refinement in a second round C6 symmetry was enforced.

For *de novo* structure prediction of the tail we used ModelAngelo with a no_seq flag [55]. The resulting prediction was used to first identify the gene in the Mystique genome using NCBI tBLASTn. The identified protein was manually built into the tail density using COOT [98] and further refined with *Phenix* [99]. A structural similarity search was done with Foldseek with default parameters. The structure prediction of Mystique head major capsid protein was done using AlphaFold2 [100] collab notebook using the sequence annotated in Mystique genome.

### *A. baumannii* phylogenetic analysis

Host genomes were downloaded from NCBI and accessed in May, 2024. A maximum parsimony tree based on whole-genome single nucleotide polymorphisms (SNPs) was constructed using kSNP4.1 [101], with a Kmer size of 17. The tree was rooted on strain 11669 (accession number GCF_006493685.1), as described in Galac *et al*. 2022 [36]. The resulting tree was visualized in R using ggtree v3.12.0 [102] and ggtreeExtra v1.14.0 [103]. To generate a chronogram from the phylogram, we utilized the chronos function in ape v5.0 [104]. We tested all available models (correlated, discrete, relaxed, and clock), and found that the tree generated under a strict clock was favoured. Using the chronogram, we calculated the phylogenetic D statistic [60] using function phylo.d from caper v1.0.3 [105]. All *A. baumannii* host genomes were analyzed with Kaptive v3.0.0 [61,106] to type the K and O loci of all hosts which Mystique infects, utilising the *A. baumannii* K locus primary reference database and A. baumannii OC locus primary reference database.

### Phage host range experiments

The initial experiment to test Mystique host range was done using plaque assays. These plaque assays on 103 highly diverse clinical *A. baumannii* strains were done by growing the bacterial strains overnight in glass microcosms containing 6 mL of LB broth while shaking at 180 rpm at 37˚C. The following day, 200 µl of each individual bacterial culture was mixed with 10 mL of 0.5% LB agar before gentle mixing and pouring on top of LB agar plates. This top layer was left to dry for approximately 30 minutes, followed by pipetting 5 µL of serial diluted phage Mystique on top of the dried top agar layer (*n* = 3 per plate). 1:10 serial dilutions of Mystique were prepared in 96-well plates, with approximately 1 x 10^9^ pfu/mL as the undiluted phage concentration. These plates were then incubated overnight at 37˚C before being checked for phage clearance the following day. Phage clearance in this case indicates any indication of a zone of clearing. This same method was used to test Mystique susceptibility for *A. nosocomialis* M2, *A. calcoaceticus* T8, and *A. baylyi* ADP1.

All phage host range and infectivity experiments in liquid were done by inoculating 60 µL from overnight bacterial cultures glass microcosms containing 6 mL of LB medium. 10^4^ pfu/mL of phage Mystique or Maestro, FG03, FG04, or CO01 were then added to the glass microcosms followed by incubation overnight at 37˚C at 180 rpm (*n* = 3 per treatment). Transfers of 1:100 into fresh LB were done daily for a total of three days, and phage titres were either tracked daily (Fig 6) or assessed by the end of the experiment (Figs 1, 2 and S3) by pipetting serial dilutions of chlorophorm-treated lysate on lawns of *A. baumannii* 43159.

Experiments to test for the impact of capsule production on Mystique infectivity were done using plaque assays of phage Mystique on lawns of *wza*::T26, *wzb*:T26, *∆wzc*, and *itrA*::T26 as well as ∆*wzc* + *wzc* and the AB5075 wild-type. Additionally, an evolution experiment tracking phage titres over time when inoculated with all isogenic mutants and the wild-type strain was done as described above.

### Silica gradients for visualising capsule production

Bacterial polysaccharide capsule production was analysed using a 12.5% gradient of colloidal silica (Ludox, Sigma Chemical) as previously described[41]. In brief, cells were grown in LB medium to an optical density A_600_ of 0.9 or on agar plates for 4 hours at 37^o^C where cells were harvested with 3 ml LB and the optical density adjusted to A_600_ of 0.9. Cells from broth or agar were pelleted and resuspended in 1 ml of 12.5% Ludox colloidal silica, followed by centrifugation in 2 ml microcentrifuge tubes at 15,000 rpm for 30 min.

### Statistics

All analyses of the effects of phage(s) on growth rates (Figs 1C, 2D, 5B and 7D) were done using linear model approaches with Tukey post hoc testing for multiple comparisons. For these, OD_600_ measurements were the response variable, with treatment, time, and replica as explanatory variables. Phage titres over time (Figs 5C and 7C) were also analysed using linear models with Tukey post hoc testing for multiple comparisons. Here, the response variable was phage titre, with treatment and replica as explanatory variables, as well as time where relevant.

Throughout the paper, pairwise comparisons were done using the Emmeans package [107], and model fits were assessed using Chi-squared tests and by comparing Akaike information criterion (AIC) values, as well as plotting residuals and probability distributions using histograms and quantile-quantile plots (Q-Q plots), respectively. All statistical analyses were done using R version 4.3.0. [108], its built-in methods, and the Tidyverse package version 2.0.0 [109].

## Supporting information

S1 Fig Measurement of capsule production of bacterial cells using silica density gradients. Comparison of the level of capsulation for AB5075 from liquid culture or a bacterial lawn.
(PDF)

S2 Fig Aggregates rapidly form in bacterial cultures when co-inoculated with phage Mystique. Photos taken of a 96-well plate after 24 hours of co-inoculation with various phages after measuring OD_600_ every 5 minutes while shaking. Mystique in particular causes aggregates that are likely to affect readings, as does phage CO01 (see Figs 1C and 2D for readings over time).
(PDF)

S3 Fig Mystique can infect both the VIR-O and AV-T states of AB5075. Plaque assays of Mystique on bacterial lawns of AB5075 in either the VIR-O or AV-T states. Strain 423159 included as control.
(PDF)

S4 Fig Comparisons of phage morphologies. TEM images showing phages Mystique, Maestro, CO01, and FG03. The image of CO01 was taken from [33]. Mystique is a siphovirus whereas Maestro, CO01, and FG03 are myoviruses.
(PDF)

S5 Fig Structure prediction of Mystique’s major capsid protein structure suggests HK97 fold. **A** AlphaFold2 structure prediction shows major structural features of HK97 fold: A- and P-domains with a characteristic backbone helix and E-loop. **B** Rigid-body fitting of the predicted structure into the experimental density forming an asymmetric unit. **C** Icosahedral symmetrisation of the asymmetric unit fills most of the capsid’s density, while unfilled densities located at trifold or pseudotrifold locations suggest an unidentified cement or decoration protein.
(PDF)

S6 Fig Structure of a Mystique’s tail monomer is highly similar to a phlagellotropic bacteriophage YSD1. **A** Side by side comparison of a YSD1 tail protein monomer (top, yellow) and Mystique phage tail protein monomer (bottom, blue). These proteins appear structurally very similar yet share very low primary sequence similarity as shown by the **B** sequence alignment of YSD1 tail protein (upper sequence) and Mystique tail protein (lower sequence). Additionally, Mystique’s tail protein lacks a C-terminal domain and has a truncated N-terminal domain. **C** Cross-section view of the tail model where residues were coloured by their electronegativity showing the negatively charged central cavity.
(PDF)

S7 Fig Mystique can infect and amplify on strains in liquid that it does not lyse on a lawn. Out of the 15 strains Mystique was unable to lyse on a bacterial lawn, six proved to be susceptible in liquid culture.
(PDF)

S8 Fig Testing for phylogenetic signal across Mystique susceptible strains. D values (black vertical lines) as a measure of phylogenetic signal, where a D value of 1 (red lines) indicates randomness and 0 (blue lines) implies departure from the randomness expected under a Brownian evolution threshold model. Calculated for *A. baumannii* strains susceptible to phage Mystique either on **A** plate or in **B** liquid (Fig 5)
(PDF)

S9 Fig Mutations in key capsule genes for all strains of *A. baumannii* in this study, compared to AB5075. The range of mutations observed in the *itrA*, *wza*, *wzb*, and *wzc* genes for all strains of *A. baumannii* which were used to assess initial Mystique host range, compared to AB5075. These genes encode important components of the capsular polysaccharide synthesis pathway, and we found mutations ranging from full deletions to no difference compared to AB5075.
(PDF)

S1 Data Detailed infection data for Mystique’s *A. baumannii* host range. Includes strain numbers, phage susceptibility, estimated phage titres, type of phage clearance, K locus, and outer core locus information.
(XLSX)
