# Supplementary figures and images for: Mystique, a broad host range Acinetobacter phage, reveals the impact of culturing conditions on phage isolation and infectivity
Source: PLoS Pathog. 2025 Apr 10;21(4):e1012986. doi: 10.1371/journal.ppat.1012986 (PMC12013898; doi:10.1371/journal.ppat.1012986)

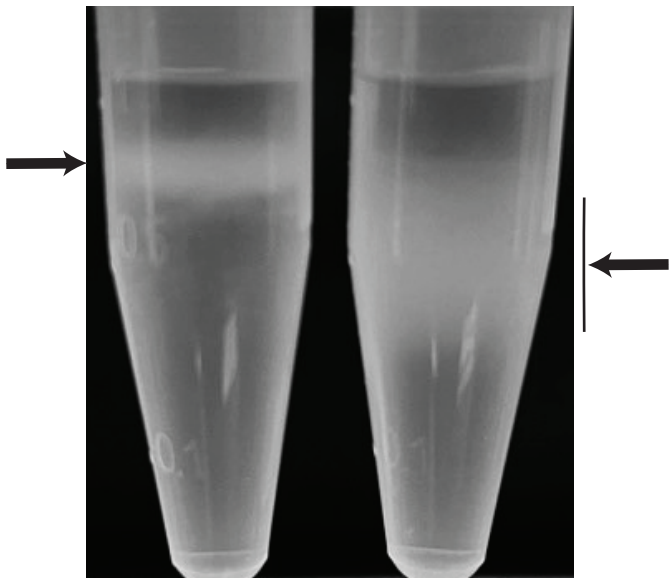

Liquid culture

Bacterial lawn

AB5075 WT

Supplement: S1 Fig — Comparison of the level of capsulation for AB5075 from liquid culture or a bacterial lawn. (PDF) [file ppat.1012986.s001.pdf]

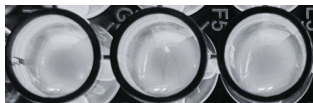

AB5075

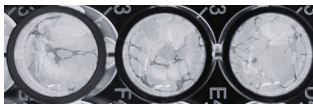

+  $\phi$ Mystique

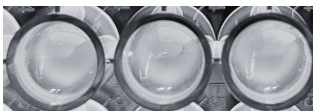

+  $\phi$ Maestro

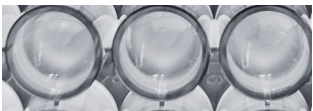

+  $\phi$ FG03

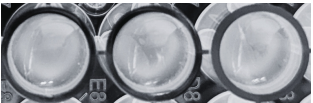

+  $\phi$ FG04

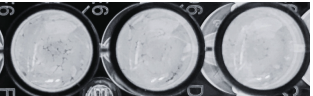

+  $\phi$ CO01

Supplement: S2 Fig — Photos taken of a 96-well plate after 24 hours of co-inoculation with various phages after measuring OD600 every 5 minutes while shaking. Mystique in particular causes aggregates that are likely to affect readings, as does phage CO01 (see Figs 1C and 2D for readings over time). (PDF) [file ppat.1012986.s002.pdf]

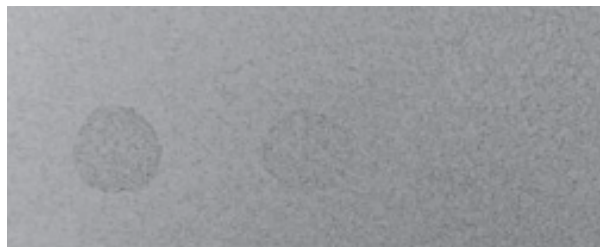

AB5075 VIR-O

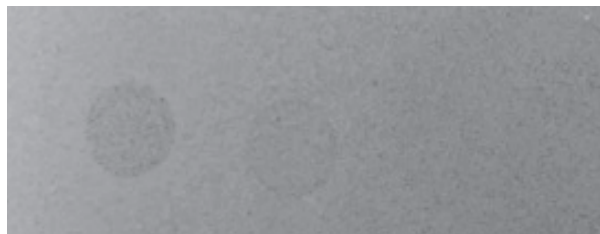

AB5075 AV-T

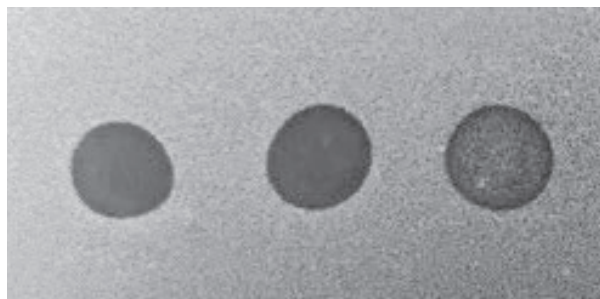

423159

Undiluted

$10^{-1}$

$10^{-2}$

Supplement: S3 Fig — Plaque assays of Mystique on bacterial lawns of AB5075 in either the VIR-O or AV-T states. Strain 423159 included as control. (PDF) [file ppat.1012986.s003.pdf]

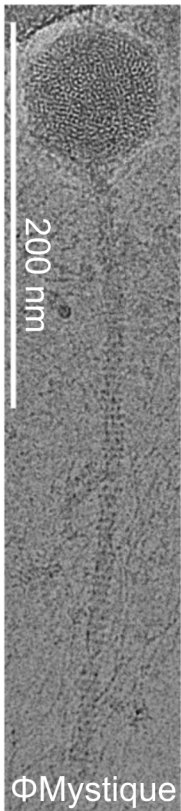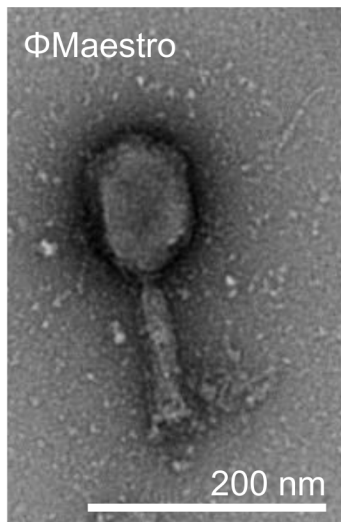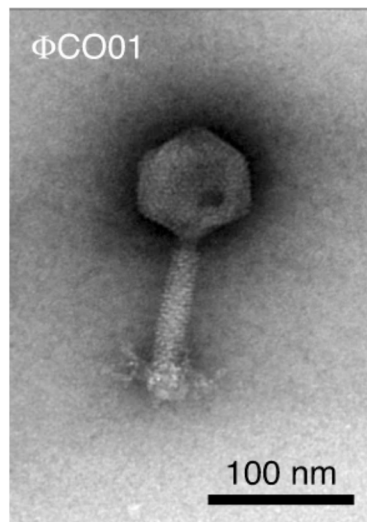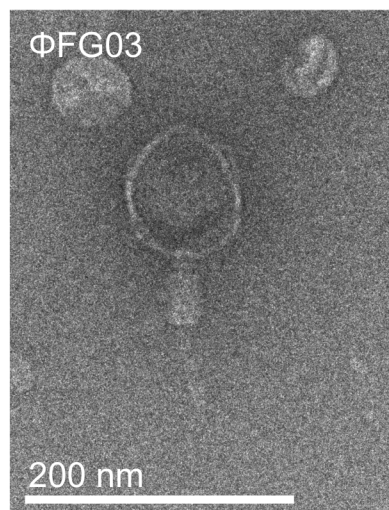

Supplement: S4 Fig — TEM images showing phages Mystique, Maestro, CO01, and FG03. The image of CO01 was taken from [33]. Mystique is a siphovirus whereas Maestro, CO01, and FG03 are myoviruses. (PDF) [file ppat.1012986.s004.pdf]

**A**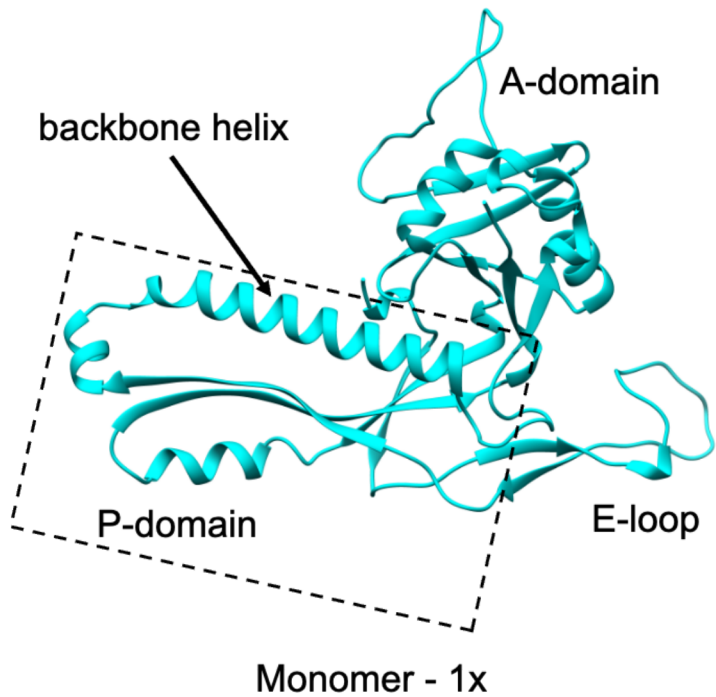**B**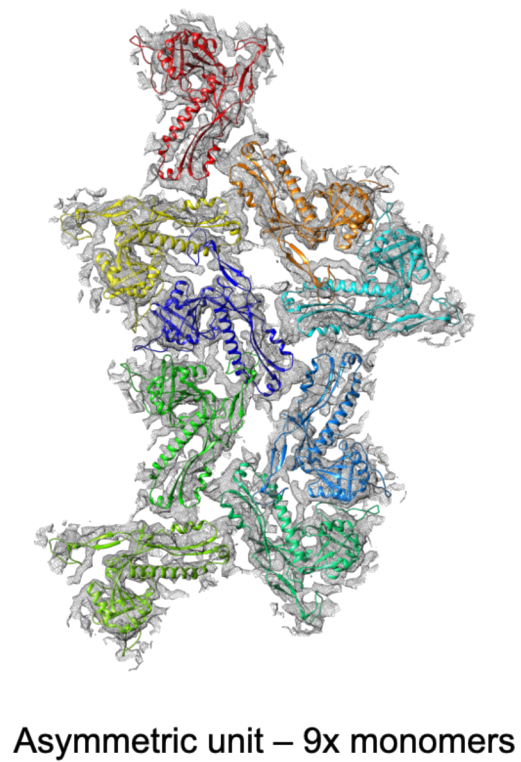**C**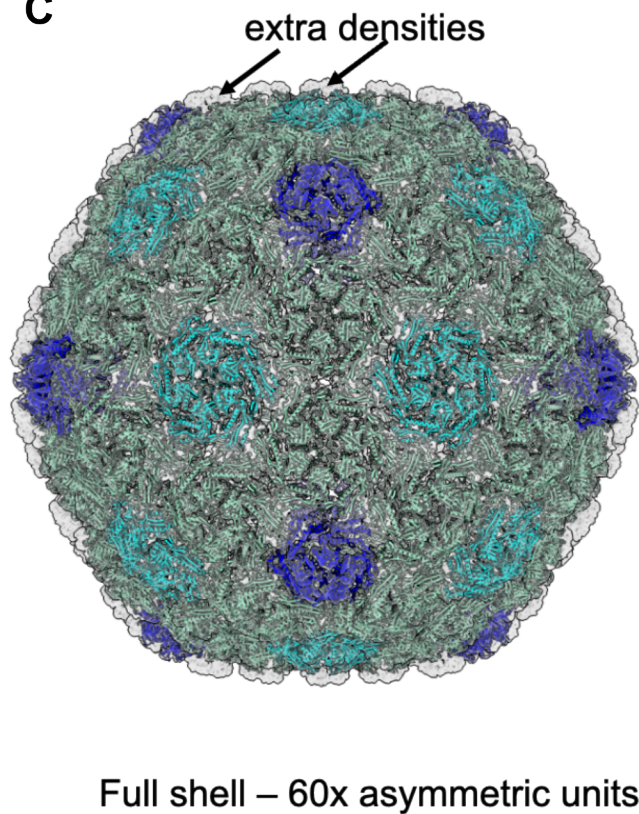

Supplement: S5 Fig — A AlphaFold2 structure prediction shows major structural features of HK97 fold: A- and P-domains with a characteristic backbone helix and E-loop. B Rigid-body fitting of the predicted structure into the experimental density forming an asymmetric unit. C Icosahedral symmetrisation of the asymmetric unit fills most of the capsid’s density, while unfilled densities located at trifold or pseudotrifold locations suggest an unidentified cement or decoration protein. (PDF) [file ppat.1012986.s005.pdf]

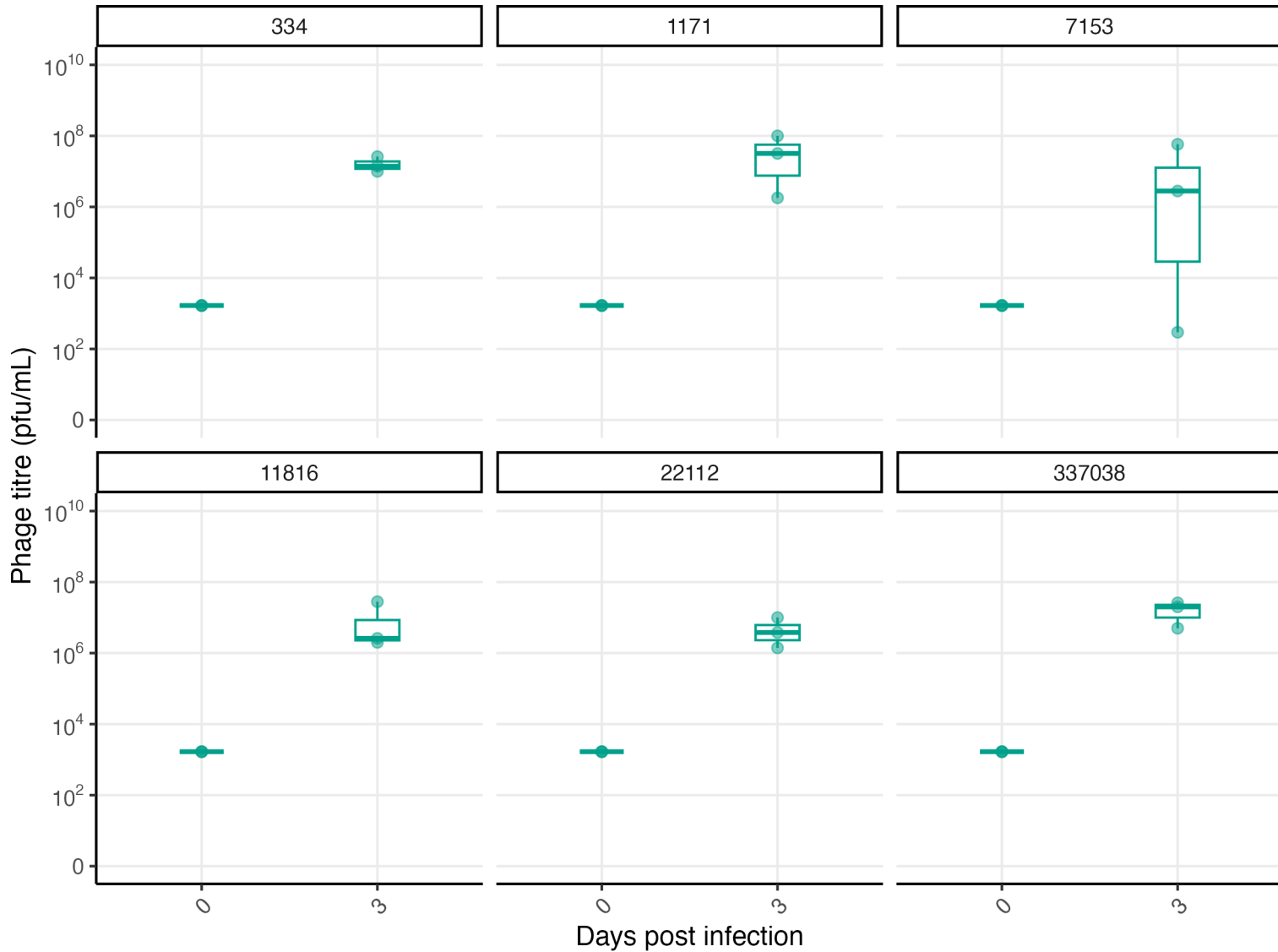

Supplement: S7 Fig — Out of the 15 strains Mystique was unable to lyse on a bacterial lawn, six proved to be susceptible in liquid culture. (PDF) [file ppat.1012986.s007.pdf]

**A**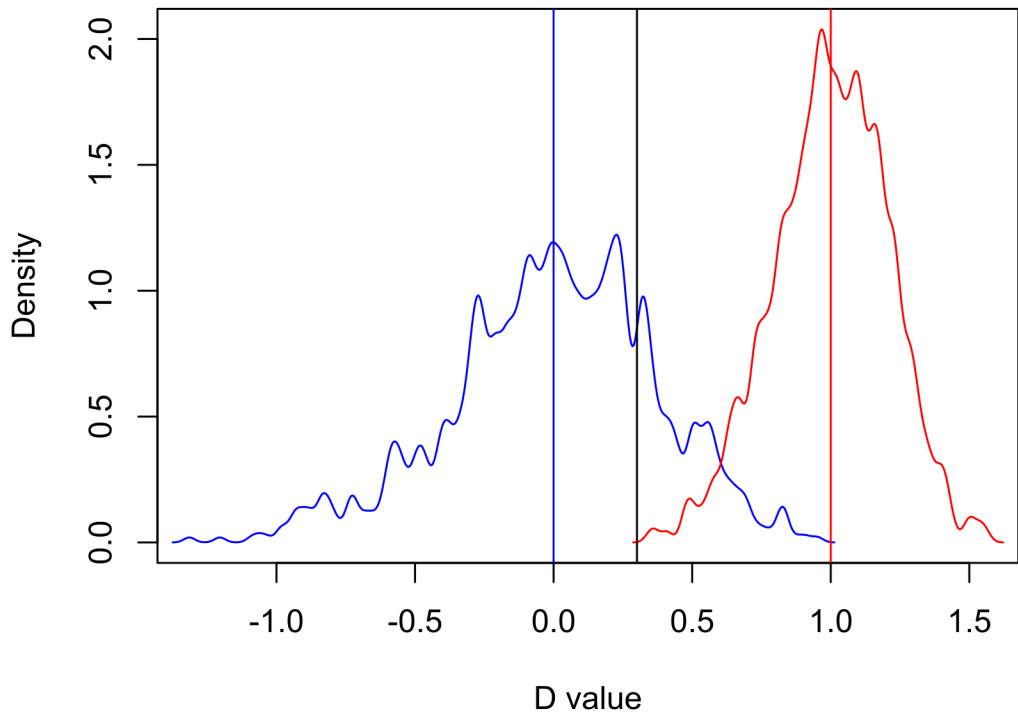**B**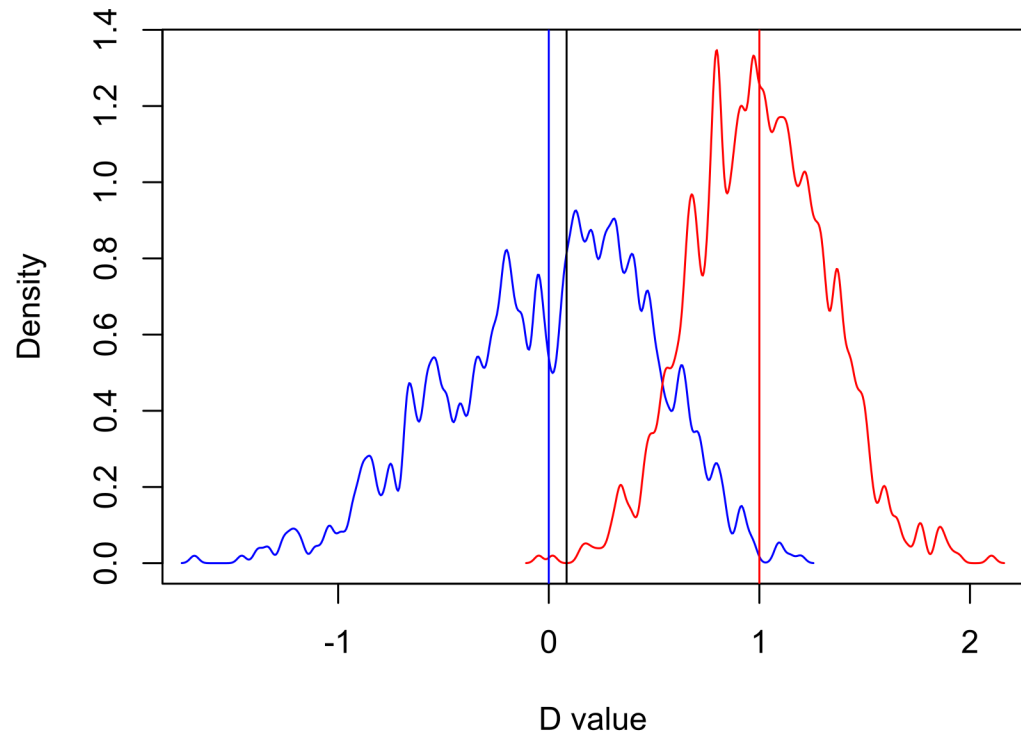

**A**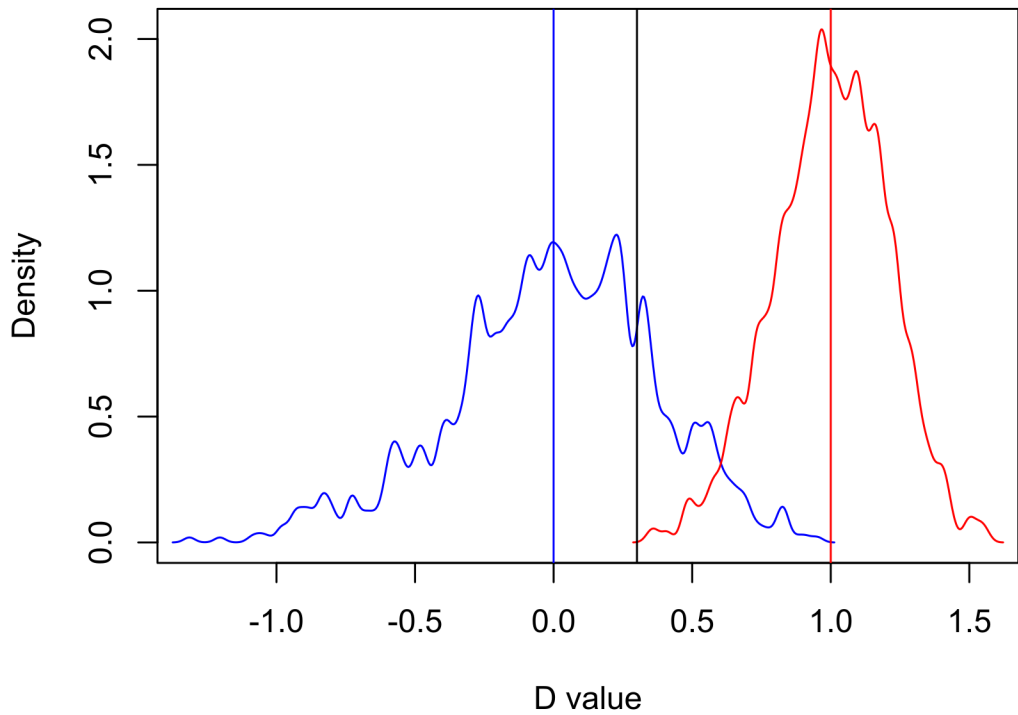**B**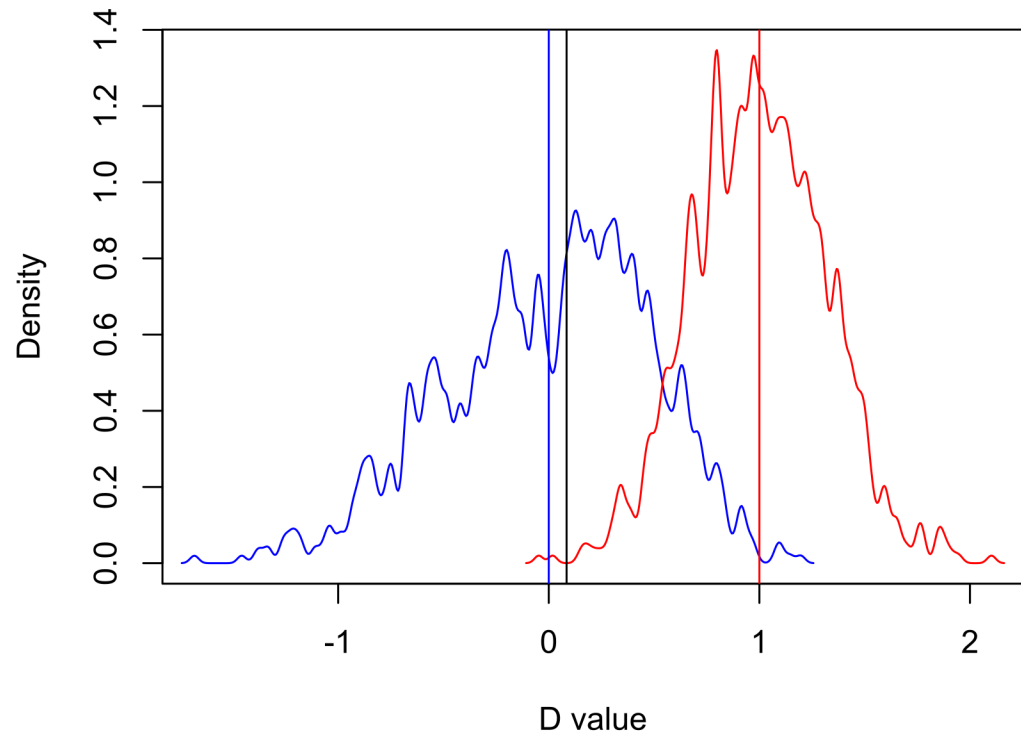

Supplement: S8 Fig — D values (black vertical lines) as a measure of phylogenetic signal, where a D value of 1 (red lines) indicates randomness and 0 (blue lines) implies departure from the randomness expected under a Brownian evolution threshold model. Calculated for A. baumannii strains susceptible to phage Mystique either on A plate or in B liquid (Fig 5) (PDF) [file ppat.1012986.s008.pdf]
