# Supplementary material for: Mystique, a broad host range Acinetobacter phage, reveals the impact of culturing conditions on phage isolation and infectivity
Source: PLoS Pathog. 2025 Apr 10;21(4):e1012986. doi: 10.1371/journal.ppat.1012986 (PMC12013898; doi:10.1371/journal.ppat.1012986)

A

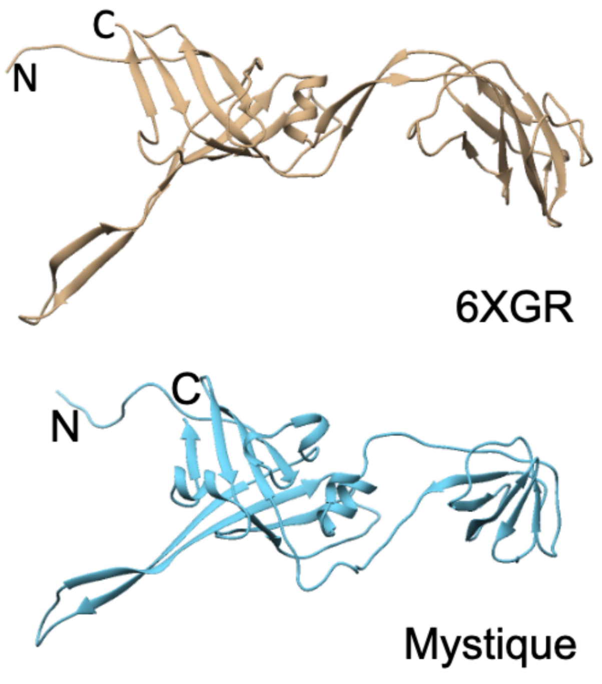

B

```
CLUSTAL O(1.2.4) multiple sequence alignment


6xgr            MNDNYQNNYVVGRGTVYFDRFQDGTNRKTGEMYFGNTPEFTINTDSETLDHYSSDHGMRV 60
Mystique_tail   MQQNF--------------------GTALGDGFVLNEATLMIGALGSALDLTEEEHSVGL 40
                *::*:                    .   *: :. *   : *.: ..:**  ..:*.: :

6xgr            MDASV------LLEASQGGTFTCDNINA-DNLALWFLGEVSNTTQTQQTDAKEVFNPIMR 113
Mystique_tail   FKNLAIANDKTFQDLNQGVT--QDTVHSQKTGDNWTISGN-----------GYEYNP--R 85
                :.  .      : : .** *   *.::: ..   * :.                :**  *

6xgr            GRYYQLGTTD--DNPTGVRGVTNFQMVKADASIA---IS------VGSGDITSIVGATVV 162
Mystique_tail   TIMYALGQAGFTADPTAARTRA---VVSAPAAVGVSEISVQSATGLAVGDWVILYNKLGD 142
                   * ** :.   :**..*  :   :*.* *::.   **      :. ** . : .

6xgr            NPAGNYEIDLEAGRIYIEPDSTDLSGNVQIAVQYDVDAQKRTLVIGK-SN----MVYGAL 217
Mystique_tail   NNGLAYKIDAIAT------NTITLDRDLVAPVAVGDELVKSTLINTNNPNSCSGAEYFSA 196
                * .  *:**  *       ::  *. ::   *  . :  * **:  :  *      * :

6xgr            RMISDNPVGLNKNYYFPKVSIAPDGDYALKGDDWQVMSFTFKAMQLNNITQRVYIDIVEA 277
Mystique_tail   KIVSADVNCNPIVVIVPKVQITSGLNLAFGATDYANIAYQMKAMALTKE--RCWL----- 249
                :::* :         .***.*: . : *: . *:  ::: :*** *.:   * ::

6xgr            AAAVDPTAQRTIEITPASTTATTGGAGVVCTVTVRDGTGTAVQGDAVTFTTVAGATVTPN 337
Mystique_tail   ------------------------------------------------------------ 249


6xgr            SATTGASGTATTTVNRTAAGTATVTATLANGKAATTGTITFSAP 381
Mystique_tail   -------------------------------------------- 249
```

C

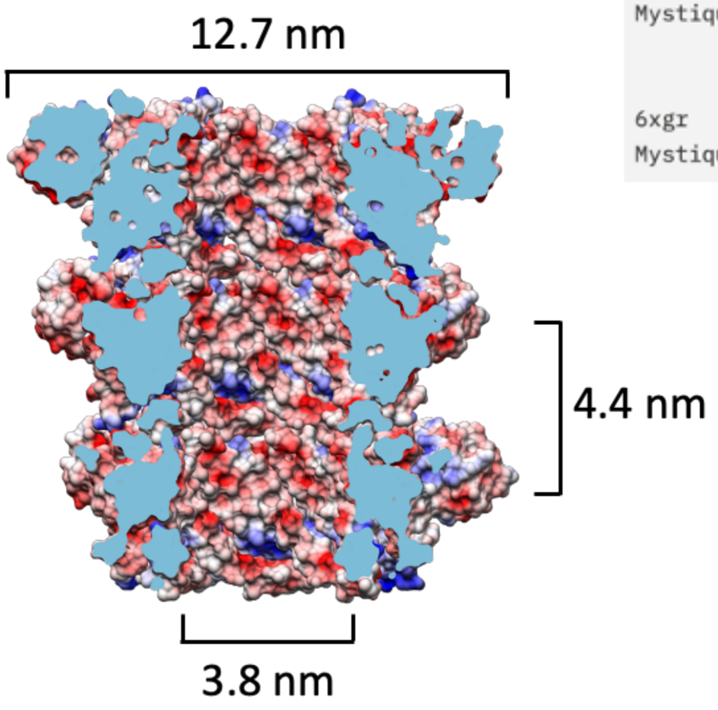

Supplement: S6 Fig — A Side by side comparison of a YSD1 tail protein monomer (top, yellow) and Mystique phage tail protein monomer (bottom, blue). These proteins appear structurally very similar yet share very low primary sequence similarity as shown by the B sequence alignment of YSD1 tail protein (upper sequence) and Mystique tail protein (lower sequence). Additionally, Mystique’s tail protein lacks a C-terminal domain and has a truncated N-terminal domain. C Cross-section view of the tail model where residues were coloured by their electronegativity showing the negatively charged central cavity. (PDF) [file ppat.1012986.s006.pdf]
