# Supplementary material for: Mystique, a broad host range Acinetobacter phage, reveals the impact of culturing conditions on phage isolation and infectivity
Source: PLoS Pathog. 2025 Apr 10;21(4):e1012986. doi: 10.1371/journal.ppat.1012986 (PMC12013898; doi:10.1371/journal.ppat.1012986)
Supplement: S9 Fig — The range of mutations observed in the itrA, wza, wzb, and wzc genes for all strains of A. baumannii which were used to assess initial Mystique host range, compared to AB5075. These genes encode important components of the capsular polysaccharide synthesis pathway, and we found mutations ranging from full deletions to no difference compared to AB5075. (PDF) [file ppat.1012986.s009.pdf]

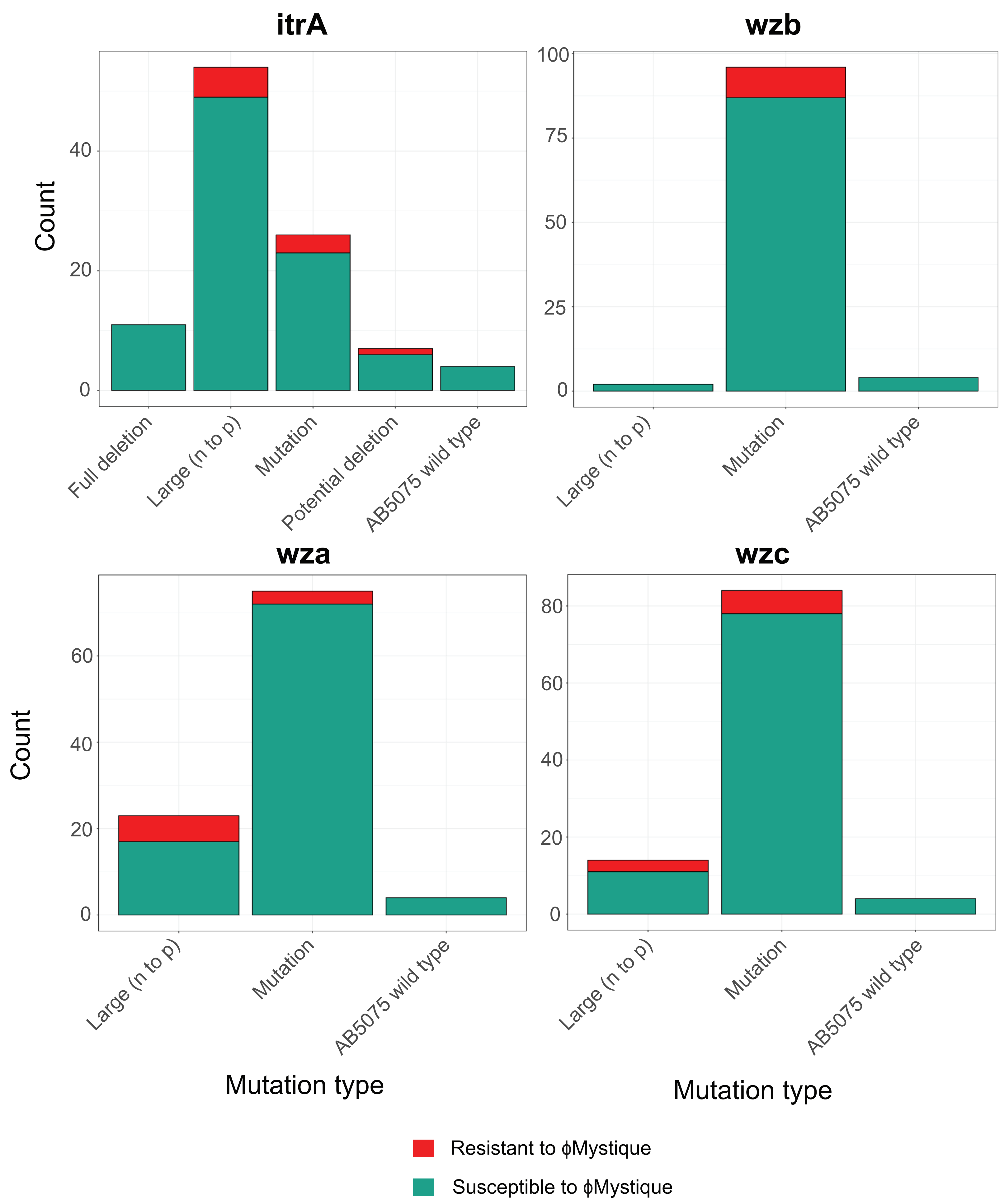
itrA
wzb
wza
wzc
Count
Count
Full deletion
Large (n to p)
Mutation
Potential deletion
AB5075 wild type
Mutation type
Mutation type
Resistant to ϕMystique
Susceptible to ϕMystique
